# Poverty-related and neglected diseases – an economic and epidemiological analysis of poverty relatedness and neglect in research and development

**DOI:** 10.3402/gha.v8.25818

**Published:** 2015-01-22

**Authors:** Peter von Philipsborn, Fridolin Steinbeis, Max E. Bender, Sadie Regmi, Peter Tinnemann

**Affiliations:** 1 Faculty of Medicine, Technische Universität München, Munich, Germany; 2 Universities Allied for Essential Medicines Europe e.V. (UAEM), Berlin, Germany; 3 Charité Universitätsmedizin Berlin, Berlin, Germany; 4 Institute for Social Medicine, Epidemiology and Health Economics, Charité Universitätsmedizin Berlin, Berlin, Germany; 5 Institute for Science, Ethics and Innovation, University of Manchester, Manchester, United Kingdom

**Keywords:** poverty-related and neglected diseases, neglected tropical diseases, research and development, disease burden, double burden, global burden of disease, research and development expenditure

## Abstract

**Background:**

Economic growth in low- and middle-income countries (LMIC) has raised interest in how disease burden patterns are related to economic development. Meanwhile, poverty-related diseases are considered to be neglected in terms of research and development (R&D).

**Objectives:**

Developing intuitive and meaningful metrics to measure how different diseases are related to poverty and neglected in the current R&D system.

**Design:**

We measured how diseases are related to economic development with the income relation factor (IRF), defined by the ratio of disability-adjusted life-years (DALYs) per 100,000 inhabitants in LMIC versus that in high-income countries. We calculated the IRF for 291 diseases and injuries and 67 risk factors included in the Global Burden of Disease Study 2010. We measured neglect in R&D with the neglect factor (NF), defined by the ratio of disease burden in DALYs (as percentage of the total global disease burden) and R&D expenditure (as percentage of total global health-related R&D expenditure) for 26 diseases.

**Results:**

The disease burden varies considerably with the level of economic development, shown by the IRF (median: 1.38; interquartile range (IQR): 0.79–6.3). Comparison of IRFs from 1990 to 2010 highlights general patterns of the global epidemiological transition. The 26 poverty-related diseases included in our analysis of neglect in R&D are responsible for 13.8% of the global disease burden, but receive only 1.34% of global health-related R&D expenditure. Within this group, the NF varies considerably (median: 19; IQR: 6–52).

**Conclusions:**

The IRF is an intuitive and meaningful metric to highlight shifts in global disease burden patterns. A large shortfall exists in global R&D spending for poverty-related and neglected diseases, with strong variations between diseases.

Over the past 20 years, rapid economic change in low and middle income countries has raised interest in how disease burden patterns are related to economic development. It is commonly held that in the course of the global epidemiological transition, a growing number LMIC face a double burden of both poverty-related, communicable diseases and affluence-related, non-communicable diseases at the same time (1, 2).

Meanwhile, poverty-related diseases are still considered to be neglected in research and development (R&D). While affluence-related diseases may attract considerable commercial R&D funding, many poverty-related diseases are considered neglected in the current R&D system (3–10). Differing assessments of the extent and relevance of this so-called R&D gap influence the broader debate on global health R&D policy (5, 11, 12). Concerns about these issues have led to negotiations on a possible Global Health R&D Convention, as proposed by the Consultative Expert Working Group on Research and Development (CEWG) commissioned by the World Health Organization (WHO), subsequent debates on a Global Health R&D Observatory, and ongoing WHO-sponsored R&D demonstration projects (5, 6, 13–17).

Based on a definition of Type I, II, and III diseases discussed in a background document prepared by the WHO Secretariat (18) and by Røttingen et al. (13), we propose an income relation factor (IRF) as a quantitative, intuitive, and meaningful metric for the degree to which diseases, disease groups, and risk factors are related to the level of economic development. Based on the IRF and work done by the WHO Secretariat (18) and Røttingen et al. (13), who used disease burden data for 2004, we propose quantitative definitions for poverty- and affluence-related diseases based on data from the Global Burden of Disease Study 2010 (GBD 2010). Moreover, we assess the size and the characteristics of the gap in R&D for a subset of poverty-related diseases by comparing global R&D expenditure and disease burden.

## Methods

**Fig. 1:**
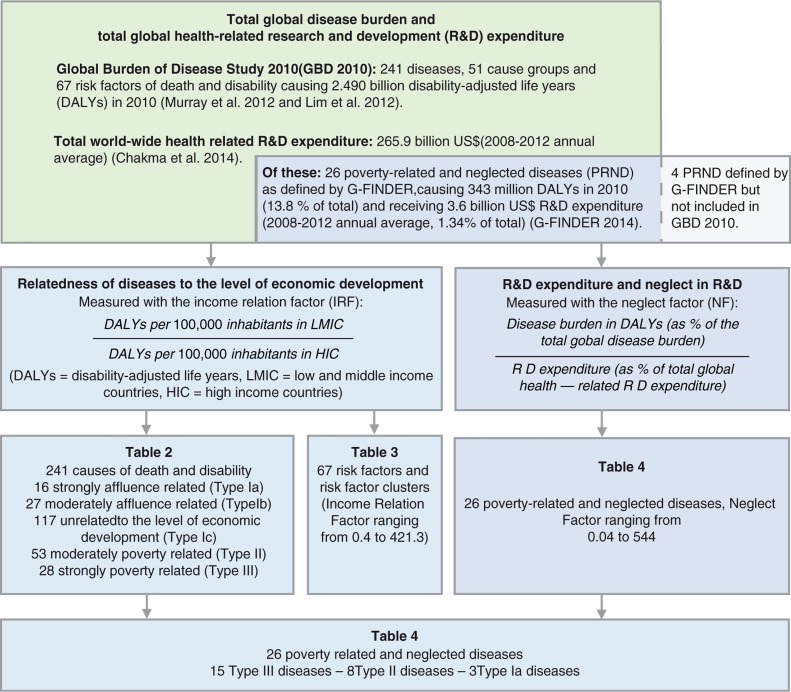
Data sources and analytical steps of our analysis of income relatedness and neglect in terms of research & development (R&D).

We conduct our analysis in two steps. First, we analyze 291 diseases, injuries and cause groups, and 67 risk factors and risk factor clusters included in the GBD 2010 (19, 20) with regard to their relatedness to the level of economic development. Second, we analyze the R&D gap for 26 diseases and disease groups commonly defined as poverty related and neglected and for which sufficiently specific R&D expenditure data were available, by comparing disease burden and R&D expenditure. An overview of our methodology is given in Fig. 1.

### Relatedness of diseases to the level of economic development

In 2001, the WHO Commission on Macroeconomics and Health proposed a scheme for classifying diseases according to their relatedness to the level of economic development which distinguishes three disease types (disease Types I, II, and III, see Table 1) (18, 21). The WHO Secretariat (18), as well as Røttingen et al. (13), propose the following ratio to operationalize this classification scheme, based on DALY figures (disability-adjusted life years, a composite figure that captures both premature mortality and the prevalence and severity of ill-health) for different world regions:

$$\frac{\text{DALYs per 100},\text{000 inhabitants in low}-\text{and middle}-\text{income countries}}{\text{DALYs per 100},\text{000 inhabitants in high}-\text{income countries}}$$

**Table 1:** The WHO classification of diseases according to their relatedness to the level of economic development and our proposed substratification and nomenclature

	Verbal definition of the WHO Commission on Macroeconomics and Health in 2001	Operationalization proposed by the WHO Secretariat (18) and Røttingen et al. (13)	Our proposed substratification and nomenclature.
Disease type I	‘Type I diseases are incident in both rich and poor countries, with large numbers of vulnerable population in both’ (21).	Diseases for which the disease burden in low- and middle-income countries (LMIC) is not more than three times higher than in high-income countries (HIC), measured in DALYs (disability-adjusted life years) per 100,000 inhabitants (13, 18).	Type Ia diseases, or strongly affluence-related diseases, with an income relation factor, IRF<0.33. Type Ib diseases, or moderately affluence-related diseases, with 0.33≥IRF<0.66. Type Ic diseases, or diseases unrelated to the level of economic development, with 0.66≥IRF<3.
Disease type II	Type II diseases ‘are incident in both rich and poor countries, but with a substantial proportion of the cases in poor countries’ (21).	Diseases for which the disease burden in LMIC is at least three but not more than 35 times higher than in HIC (13, 18).	Type II diseases, or moderately poverty-related diseases, with 3≥IRF<35 (13, 18).
Disease type III	Type III diseases are diseases ‘that are overwhelmingly or exclusively incident in developing countries’ (21).	Diseases for which the disease burden in LMIC is more than 35 times higher than in HIC (13, 18).	Type II diseases, or strongly poverty-related diseases, with IRF≥35 (13, 18).

We use the term income relation factor (IRF) for this ratio. We define Type III diseases (IRF≥35) as strongly poverty related, and Type II diseases (3≥IRF<35) as moderately poverty related. To accommodate all conditions, and not just those related to poverty, we expand the existing classification by subdividing the group of Type I diseases (0≥IRF<3) into three groups: conditions unrelated to economic development (Type Ic, 0.66≥IRF<3), moderately affluence-related conditions (Type Ib, 0.33≥IRF<0.66), and strongly affluence-related conditions (Type Ia, IRF<0.33). We then calculate the IRF for all diseases, injuries and cause groups included in the GBD 2010 and categorize them according to the above definitions. An overview of the different disease categories and their definitions is given in Table 1. Based on DALYs attributable to the independent effects of risk factors and risk factor clusters as estimated by the GBD 2010, we calculate the IRF for the 67 risk factors and risk factor clusters included in the GBD 2010 (20).

### R&D expenditure and neglect in R&D

In the term neglected diseases, the word neglect refers to the notion that for the diseases in question, the proportion of current R&D efforts is considered to fall short of the proportion which would be equitable and efficient under the given conditions. In our operationalization of neglect, we use R&D expenditure as a yardstick for current R&D efforts, as it reflects both the commercial interest of industry and the political commitment of governments and philanthropy. We use the contribution of diseases to the global disease burden measured in DALYs as an incomplete and controversial proxy for the proportion of R&D efforts which should ideally be directed toward specific diseases. To quantify the magnitude of the imbalance between R&D efforts and assumed R&D needs for specific diseases – that is, the size of the R&D Gap – we calculate the R&D expenditure in US$ per DALY for individual diseases and disease groups. This metric has been used in previous studies on neglect in R&D (6, 13, 22, 23). In addition, we propose the neglect factor (NF) as a new summary measure, with the following definition:

$$\text{Neglect Factor}=\frac{\text{Disease burden in DALYs}\left(\text{in}\%\text{of the total global disease burden}\right)}{\text{R}\&\text{D expenditure}(\text{in}\%\text{of total global health}-\text{related R}\&\text{D expenditure})}$$

For R&D expenditure, we used data provided by G-FINDER (Global Funding of Innovation for Neglected Diseases) (23, 24) and Chakma et al. (25). G-FINDER includes comprehensive R&D expenditure data on 30 diseases fulfilling three criteria: 1) the disease disproportionately affects people in developing countries; 2) there is a need for new pharmaceutical products; and 3) there is a market failure, that is, a commercial market insufficient to attract industry R&D (23). Of these 30 diseases, we exclude four diseases which are not included in GBD 2010, and for which we therefore lack comparable disease burden data (see methodological appendix, Supplementary file 1, for details).

### Development and regional distribution of the burden of poverty-related and neglected diseases

To track the public health relevance of poverty-related and neglected diseases (PRND) over time, and to understand possible reasons for their neglect, we analyze the development of the disease burden caused by PRND over the past 20 years, and the geographical distribution of the disease burden over the 21 regions covered in GBD 2010. For all figures based on GBD 2010 data, we calculate 95% uncertainty intervals based on the 95% uncertainty intervals as reported in GBD 2010 (please refer to the methodological appendix, Supplementary file 1, for details).

## Results

### Relatedness of diseases, injuries, and risk factors to the level of economic development

In 2010, the overall IRF for all causes was 1.4, indicating an approximately 1.4 times higher total disease burden per capita in LMIC compared to high-income countries (HIC). This is a decrease from 1990, when the overall IRF was 1.8. The relatedness of major disease groups to the level of economic development is presented in Table 2. Data for all 291 diseases, injuries, and cause groups included in the GBD 2010 are presented in Supplementary file 2. Table 3 presents the IRF for the main risk factor clusters analyzed in GBD 2010 (data on all 67 risk factors are provided in Supplementary file 3). In 2010, the disease burden attributable to risk factors associated with unimproved water and sanitation, as well as child and maternal undernutrition, was much higher in LMIC than in HIC (IRF 107 and 34, respectively). By contrast, the disease burden attributable to dietary risk factors, physical inactivity, physiological risk factors, alcohol and drug use, and tobacco smoking was 10–20% higher in HIC than in LMIC, resulting in an IRF between 0.9 and 0.8 (up from 0.7 to 0.6 in 1990).

**Table 2:** Relatedness of disease groups to the level of economic development

	2010				1990			
	
	DALYs per 100,000 inhabitants				DALYs per 100,000 inhabitants			
						
Cause or cause group	LMIC	HIC	IRF	Disease type	LMIC	HIC	IRF	Disease type
**All causes**	**38,041 (27,279–52,476)**	**26,421 (19,917–34,930)**	**1.4 (0.8–2.6)**	**Ic (Ic-Ic)**	**51,463 (36,115–73,296)**	**28,629 (22,174–36,789)**	**1.8 (1.0–3.3)**	**Ic (Ic-II)**
**Communicable, maternal, neonatal, and nutritional disorders**	**14,604 (10,322–20,676)**	**1,416 (1,084–1,869)**	**10.3 (5.5–19.1)**	**II (II-II)**	**26,602 (18,241–39,060)**	**2,084 (1,628–2,716)**	**12.8 (6.7–24.0)**	**II (II-II)**
HIV/AIDS and tuberculosis	2,214 (1,842–2,581)	150 (132–171)	14.7 (10.8–19.6)	II (II-II)	1,746 (1,444–2,160)	343 (305–384)	5.1 (3.8–7.1)	II (II-II)
Diarrhea, lower respiratory infections, meningitis, and other common infectious diseases	4,720 (3,173–7,104)	695 (534–921)	6.8 (3.4–13.3)	II (II-II)	12,252 (8,029–19,289)	840 (644–1,139)	14.6 (7.0–29.9)	II (II-II)
Neglected tropical diseases and malaria	1,856 (1,200–2,863)	25 (13–45)	74.8 (26.4–224.2)	III (II-III)	2,367 (1,507–3,760)	38 (19–69)	62.8 (21.9–194.3)	III (II-III)
Maternal disorders	273 (206–365)	14 (8–24)	19.9 (8.5–44.9)	II (II-III)	490 (385–627)	16 (12–23)	30.2 (16.7–50.7)	II (II-III)
Neonatal disorders	3,394 (2,455–4,644)	354 (283–443)	9.6 (5.5–16.4)	II (II-II)	6,134 (4,350–8,216)	624 (493–773)	9.8 (5.6–16.7)	II (II-II)
Nutritional deficiencies	1,440 (1,013–1,997)	118 (78–173)	12.2 (5.9–25.5)	II (II-II)	2,527 (1,869–3,396)	148 (104–209)	17.0 (8.9–32.5)	II (II-II)
Other communicable, maternal, neonatal, and nutritional disorders	707 (433–1,122)	61 (36–92)	11.6 (4.7–31.0)	II (II-II)	1,087 (657–1.612)	75 (49–118)	14.5 (5.6–33.0)	II (II-II)
**Non-communicable diseases**	**19,079 (13,870–25,683)**	**22,470 (16,895–29,815)**	**0.8 (0.5–1.5)**	**Ic (Ib-Ic)**	**19,841 (14,360–27,282)**	**23,442 (18,081–30,168)**	**0.8 (0.5–1.5)**	**Ic (Ib-Ic)**
Neoplasms	2,461 (1,783–3,157)	4,392 (3,401–5,596)	0.6 (0.3–0.9)	Ib (Ia-Ic)	2,459 (1,827–3,192)	4,575 (3,636–5,773)	0.5 (0.3–0.9)	Ib (Ia-Ic)
Cardiovascular and circulatory diseases	4,301 (3,639–4,986)	4,290 (3,877–4,973)	1.0 (0.7–1.3)	Ic (Ic-Ic)	4,334 (3,670–5,161)	5,774 (5,259–6,239)	0.8 (0.6–1.0)	Ic (Ib-Ic)
Chronic respiratory diseases	1,777 (1,386–2,299)	1,387 (1,068–1,795)	1.3 (0.8–2.2)	Ic (Ic-Ic)	2,431 (1,933–3,114)	1,453 (1,148–1,853)	1.7 (1.0–2.7)	Ic (Ic-Ic)
Cirrhosis of the liver	462 (347–601)	394 (320–465)	1.2 (0.7–1.9)	Ic (Ic-Ic)	462 (359–577)	464 (388–546)	1.0 (0.7–1.5)	Ic (Ib-Ic)
Digestive diseases except cirrhosis	491 (354–700)	391 (287–553)	1.3 (0.6–2.4)	Ic (Ib-Ic)	680 (478–922)	435 (325–601)	1.6 (0.8–2.8)	Ic (Ic-Ic)
Neurological disorders	1,029 (710–1,443)	1,342 (982–1,733)	0.8 (0.4–1.5)	Ic (Ib-Ic)	909 (612–1,294)	1,007 (743–1,324)	0.9 (0.5–1.7)	Ic (Ib-Ic)
Other mental and behavioral disorders	20 (13–30)	30 (19–44)	0.7 (0.3–1.6)	Ic (Ia-Ic)	21 (12–31)	26 (16–38)	0.8 (0.3–1.9)	Ic (Ia-Ic)
Diabetes, urogenital, blood, and endocrine diseases	1,761 (1,255–2,495)	1,916 (1,380–2,780)	0.9 (0.5–1.8)	Ic (Ib-Ic)	1,626 (1,098–2,536)	1,577 (1,152–2,211)	1.0 (0.5–2.2)	Ic (Ib-Ic)
Musculoskeletal disorders	2,247 (1,577–3,068)	3,762 (2,639–5,084)	0.6 (0.3–1.2)	Ib (Ia-Ic)	1,967 (1,364–2,690)	3,447 (2,414–4,664)	0.6 (0.3–1.1)	Ib (Ia-Ic)
Other non-communicable diseases	1,930 (1,092–3,180)	1,441 (787–2,443)	1.3 (0.4–4.0)	Ic (Ib-II)	2,504 (1,401–4,232)	1,705 (974–2,759)	1.5 (0.5–4.3)	Ic (Ib-II)
**Injuries**	**4,358 (3,087–6,117)**	**2,535 (1,937–3,246)**	**1.7 (1.0–3.2)**	**Ic (Ic-II)**	**5,020 (3,514–6,954)**	**3,104 (2,466–3,905)**	**1.6 (0.9–2.8)**	**Ic (Ic-Ic)**
Transport injuries	1,295 (946–1,773)	701 (570–874)	1.8 (1.1–3.1)	Ic (Ic-II)	1,200 (797–1,705)	1,060 (873–1,278)	1.1 (0.6–2.0)	Ic (Ib-Ic)
Unintentional injuries other than transport injuries	1,872 (1,351–2,549)	1,128 (850–1,482)	1.7 (0.9–3.0)	Ic (Ic-Ic)	2,722 (1,949–3,725)	1,182 (922–1,517)	2.3 (1.3–4.0)	Ic (Ic-II)
Self-harm and interpersonal violence	948 (647–1,315)	690 (508–865)	1.4 (0.7–2.6)	Ic (Ic-Ic)	957 (670–1,300)	848 (663–1,088)	1.1 (0.6–2.0)	Ic (Ib-Ic)
Forces of nature, war, and legal intervention	243 (142–481)	17 (10–26)	14.7 (5.6–46.2)	II (II-III)	141 (98–223)	14 (8–22)	10.2 (4.4–27.1)	II (II-II)

Own calculations based on Global Burden of Disease Study 2010 data. DALYs: disability-adjusted life years; LMIC: low- and middle-income countries; HIC: high-income countries; IRF: income relation factor, ratio of DALYs per 100,000 inhabitants in LMIC versus HIC; Disease Type Ia: strongly affluence-related diseases; Ib: moderately affluence related; Ic: unrelated to the level of economic development; II: moderately poverty related; III: strongly poverty related. In brackets, the 95% uncertainty interval is given, based on GBD 2010 figures. For details, please refer to the methodological appendix.

**Table 3:** Relatedness of risk factors and risk factor clusters to the level of economic development

	2010				1990			
	
	DALYs per 100,000 inhabitants				DALYs per 100,000 inhabitants			
						
Risk factor or risk factor cluster	LMIC	HIC	IRF	Risk factor type	LMIC	HIC	IRF	Risk factor type
**Unimproved water and sanitation**	**362 (15–708)**	**3 (0–8)**	**106.8 (2.0–5146.9)**	**III (Ic-III)**	**1,191 (61–2,175)**	**9 (0–19)**	**137.9 (3.3–6081.3)**	**III (II-III)**
Unimproved water source	133 (8–281)	2 (0–3)	85.5 (2.4–3097.8)	III (Ic-III)	483 (33–972)	4 (0–9)	121.5 (3.8–4005.0)	III (II-III)
Unimproved sanitation	255 (6–516)	2 (0–4)	137.4 (1.5–11347.7)	III (Ic-III)	824 (25–1,559)	5 (0–11)	168.9 (2.3–13871.5)	III (Ic-III)
**Air pollution**	**2,649 (2,219–3,131)**	**590 (497–699)**	**4.5 (3.2–6.3)**	**II (II-II)**	**4,776 (3,961–5,601)**	**1,179 (1,011–1,341)**	**4.0 (3.0–5.5)**	**II (Ic-II)**
Ambient particulate matter pollution	1,203 (1,032–1,387)	577 (491–668)	2.1 (1.5–2.8)	Ic (Ic-Ic)	1,643 (1,366–1,946)	1,112 (973–1,248)	1.5 (1.1–2.0)	Ic (Ic-Ic)
Household air pollution from solid fuels	1,848 (1,397–2,358)	7 (0–27)	279.7 (52.1–74103.8)	III (III-III)	3,888 (3,038–4,705)	79 (26–126)	49.1 (24.1–183.5)	III (II-III)
Ambient ozone pollution	39 (13–68)	17 (6–32)	2.3 (0.4–12.1)	Ic (Ib-II)	52 (17–95)	28 (10–47)	1.9 (0.4–9.8)	Ic (Ib-II)
**Other environmental risks**	**240 (175–323)**	**197 (135–276)**	**1.2 (0.6–2.4)**	**Ic (Ib-Ic)**	**107 (80–139)**	**77 (61–97)**	**1.4 (0.8–2.3)**	**Ic (Ic-Ic)**
Residential radon	26 (3–72)	58 (8–131)	0.5 (0.0–9.5)	Ib (Ia-II)	Not assessed for 1990 because of absence of exposure data			
Lead exposure	214 (158–280)	139 (110–174)	1.5 (0.9–2.5)	Ic (Ic-Ic)	107 (80–139)	77 (61–97)	1.4 (0.8–2.3)	Ic (Ic-Ic)
**Child and maternal undernutrition**	**2,827 (2,283–3,477)**	**83 (59–118)**	**33.9 (19.3–58.8)**	**II (II-III)**	**7,748 (6,328–9,497)**	**134 (99–176)**	**58.0 (35.9–96.0)**	**III (III-III)**
Suboptimal breastfeeding	812 (474–1,213)	5 (3–8)	160.8 (58.4–475.8)	III (III-III)	2,518 (1,498–3,630)	21 (11–32)	122.6 (47.6–330.3)	III (III-III)
Childhood underweight	1,322 (1,040–1,674)	3 (2–5)	421.3 (217.2–792.6)	III (III-III)	4,520 (3,690–5,630)	12 (8–17)	362.5 (215.3–682.5)	III (III-III)
Iron deficiency	813 (564–1,147)	70 (47–103)	11.6 (5.5–24.4)	II (II-II)	1,167 (836–1,620)	91 (66–128)	12.8 (6.5–24.5)	II (II-II)
Vitamin A deficiency	184 (91–310)	1 (0–3)	149.6 (36.2–716.9)	III (III-III)	692 (321–1,287)	3 (1–6)	240.8 (54.7–1268.6)	III (III-III)
Zinc deficiency	156 (37–301)	4 (2–8)	35.3 (4.5–148.3)	III (II-III)	556 (113–1,073)	8 (4–15)	69.2 (7.7–287.7)	III (II-III)
**Tobacco smoking. including secondhand smoke**	**2,208 (1,853–2,540)**	**2,729 (2,376–3,072)**	**0.8 (0.6–1.1)**	**Ic (Ib-Ic)**	**2,722 (2,306–3,179)**	**3,696 (3,356–4,070)**	**0.7 (0.6–0.9)**	**Ic (Ib-Ic)**
Tobacco smoking	1,887 (1,546–2,204)	2,614 (2,252–2,957)	0.7 (0.5–1.0)	Ic (Ib-Ic)	1,899 (1,572–2,288)	3,463 (3,112–3,852)	0.5 (0.4–0.7)	Ib (Ib-Ic)
Secondhand smoke	321 (233–417)	115 (76–161)	2.8 (1.4–5.5)	Ic (Ic-II)	823 (591–1,068)	233 (165–300)	3.5 (2.0–6.5)	II (Ic-II)
**Alcohol and drug use**	**2,201 (1,901–2,547)**	**2,718 (2,448–2,997)**	**0.8 (0.6–1.0)**	**Ic (Ib-Ic)**	**2,039 (1,765–2,371)**	**3,376 (3,090–3,688)**	**0.6 (0.5–0.8)**	**Ib (Ib-Ic)**
Alcohol use	1,890 (1,630–2,199)	2,208 (2,015–2,422)	0.9 (0.7–1.1)	Ic (Ic-Ic)	1,793 (1,555–2,094)	2,919 (2,686–3,179)	0.6 (0.5–0.8)	Ib (Ib-Ic)
Drug use	316 (228–431)	527 (404–674)	0.6 (0.3–1.1)	Ib (Ib-Ic)	250 (178–353)	480 (370–617)	0.5 (0.3–1.0)	Ib (Ia-Ic)
**Physiological risk factors**	**4,149 (3,766–4,519)**	**4,764 (4,373–5,215)**	**0.9 (0.7–1.0)**	**Ic (Ic-Ic)**	**3,608 (3,314–3,937)**	**5,793 (5,426–6,145)**	**0.6 (0.5–0.7)**	**Ib (Ib-Ic)**
High fasting plasma glucose	1,302 (1,030–1,592)	1,269 (1,034–1,531)	1.0 (0.7–1.5)	Ic (Ic-Ic)	1,029 (828–1,244)	1,284 (1,057–1,526)	0.8 (0.5–1.2)	Ic (Ib-Ic)
High total cholesterol	553 (328–792)	843 (651–1,059)	0.7 (0.3–1.2)	Ib (Ia-Ic)	590 (441–747)	1,551 (1,310–1,809)	0.4 (0.2–0.6)	Ib (Ia-Ib)
High blood pressure	2,571 (2,213–2,915)	2,290 (1,922–2,654)	1.1 (0.8–1.5)	Ic (Ic-Ic)	2,410 (2,120–2,706)	3,570 (3,235–3,897)	0.7 (0.5–0.8)	Ic (Ib-Ic)
High body mass index	1,198 (931–1,485)	2,317 (1,969–2,692)	0.5 (0.3–0.8)	Ib (Ib-Ic)	756 (565–964)	2,089 (1,733–2,449)	0.4 (0.2–0.6)	Ib (Ia-Ib)
Low bone mineral density	67 (48–86)	130 (95–172)	0.5 (0.3–0.9)	Ib (Ia-Ic)	52 (41–67)	95 (71–124)	0.5 (0.3–0.9)	Ib (Ia-Ic)
**Dietary risk factors and physical inactivity**	**3,669 (3,347–3,977)**	**3,924 (3,639–4,248)**	**0.9 (0.8–1.1)**	**Ic (Ic-Ic)**	**3,132 (2,840–3,435)**	**4,550 (4,194–4,859)**	**0.7 (0.6–0.8)**	**Ic (Ib-Ic)**
Diet low in fruits	1,582 (1,210–1,914)	1,141 (862–1,415)	1.4 (0.9–2.2)	Ic (Ic-Ic)	1,521 (1,168–1,848)	1,575 (1,168–1,946)	1.0 (0.6–1.6)	Ic (Ib-Ic)
Diet low in vegetables	568 (357–778)	530 (362–693)	1.1 (0.5–2.2)	Ic (Ib-Ic)	564 (350–778)	776 (526–1,014)	0.7 (0.3–1.5)	Ic (Ib-Ic)
Diet low in whole grains	621 (481–755)	440 (336–539)	1.4 (0.9–2.2)	Ic (Ic-Ic)	559 (432–685)	559 (425–688)	1.0 (0.6–1.6)	Ic (Ib-Ic)
Diet low in nuts and seeds	730 (468–956)	846 (538–1,101)	0.9 (0.4–1.8)	Ic (Ib-Ic)	666 (429–867)	1,289 (833–1,656)	0.5 (0.3–1.0)	Ib (Ia-Ic)
Diet low in milk	25 (7–43)	62 (18–105)	0.4 (0.1–2.4)	Ib (Ia-Ic)	23 (7–38)	60 (18–101)	0.4 (0.1–2.2)	Ib (Ia-Ic)
Diet high in red meat	22 (10–38)	55 (26–86)	0.4 (0.1–1.5)	Ib (Ia-Ic)	17 (7–28)	53 (25–83)	0.3 (0.1–1.1)	Ia (Ia-Ic)
Diet high in processed meat	271 (82–483)	499 (182–804)	0.5 (0.1–2.6)	Ib (Ia-Ic)	261 (73–465)	669 (201–1,123)	0.4 (0.1–2.3)	Ib (Ia-Ic)
Diet high in sugar-sweetened beverages	126 (72–199)	119 (73–182)	1.1 (0.4–2.7)	Ic (Ib-Ic)	101 (52–168)	114 (68–179)	0.9 (0.3–2.5)	Ic (Ia-Ic)
Diet low in fiber	228 (95–371)	306 (149–468)	0.7 (0.2–2.5)	Ic (Ia-Ic)	215 (92–347)	444 (204–689)	0.5 (0.1–1.7)	Ib (Ia-Ic)
Diet low in calcium	33 (23–43)	68 (44–94)	0.5 (0.3–1.0)	Ib (Ia-Ic)	29 (21–38)	62 (41–85)	0.5 (0.2–0.9)	Ib (Ia-Ic)
Diet low in seafood omega-3 fatty acids	412 (294–528)	406 (291–532)	1.0 (0.6–1.8)	Ic (Ib-Ic)	368 (266–474)	636 (465–807)	0.6 (0.3–1.0)	Ib (Ia-Ic)
Diet low in polyunsaturated fatty acids	163 (75–252)	214 (103–324)	0.8 (0.2–2.5)	Ic (Ia-Ic)	159 (75–244)	334 (160–512)	0.5 (0.1–1.5)	Ib (Ia-Ic)
Diet high in trans fatty acids	154 (108–202)	254 (184–330)	0.6 (0.3–1.1)	Ib (Ia-Ic)	107 (74–142)	383 (273–492)	0.3 (0.2–0.5)	Ia (Ia-Ib)
Diet high in sodium	907 (583–1,212)	806 (512–1,080)	1.1 (0.5–2.4)	Ic (Ib-Ic)	844 (542–1,125)	1,046 (665–1,403)	0.8 (0.4–1.7)	Ic (Ib-Ic)
Physical inactivity and low physical activity	958 (793–1,134)	1,311 (1,126–1,510)	0.7 (0.5–1.0)	Ic (Ib-Ic)	Not assessed for 1990 because of absence of exposure data			
**Occupational risk factors**	**984 (740–1,272)**	**491 (382–623)**	**2.0 (1.2–3.3)**	**Ic (Ic-II)**	**1,128 (862–1,420)**	**686 (565–831)**	**1.6 (1.0–2.5)**	**Ic (Ic-Ic)**
Occupational carcinogens	35 (20–52)	62 (44–83)	0.6 (0.2–1.2)	Ib (Ia-Ic)	26 (16–41)	68 (51–93)	0.4 (0.2–0.8)	Ib (Ia-Ic)
Occupational asthmagens	31 (20–50)	21 (14–31)	1.5 (0.7–3.7)	Ic (Ib-II)	43 (26–74)	26 (18–38)	1.6 (0.7–4.1)	Ic (Ic-II)
Occupational particulate matter, gases, and fumes	150 (70–237)	38 (14–66)	4.0 (1.1–16.5)	II (Ic-II)	210 (98–327)	41 (16–70)	5.1 (1.4–20.4)	II (Ic-II)
Occupational noise	56 (33–90)	19 (11–31)	3.0 (1.1–8.4)	Ic (Ic-II)	60 (35–97)	26 (15–44)	2.3 (0.8–6.4)	Ic (Ic-II)
Occupational risk factors for injuries	382 (250–574)	112 (93–140)	3.4 (1.8–6.2)	II (Ic-II)	434 (285–629)	260 (220–302)	1.7 (0.9–2.9)	Ic (Ic-Ic)
Occupational low back pain	330 (214–476)	240 (156–347)	1.4 (0.6–3.1)	Ic (Ib-II)	355 (228–513)	264 (173–376)	1.3 (0.6–3.0)	Ic (Ib-Ic)
**Sexual abuse and violence**	**353 (245–482)**	**286 (212–379)**	**1.2 (0.6–2.3)**	**Ic (Ib-Ic)**				
Childhood sexual abuse	110 (78–150)	136 (102–176)	0.8 (0.4–1.5)	Ic (Ib-Ic)	Not assessed for 1990 because of absence of exposure data			
Intimate partner violence	259 (162–380)	161 (101–240)	1.6 (0.7–3.8)	Ic (Ic-II)				

Own calculations based on Global Burden of Disease Study 2010 data. DALYs: disability-adjusted life years; LMIC: low- and middle-income countries; HIC: high-income countries; IRF: income relation factor, ratio of DALYs per 100,000 inhabitants in LMIC versus HIC; Risk factor Type Ia: strongly affluence-related risk factors; Ib: moderately affluence related; Ic: unrelated to the level of economic development; II: moderately poverty related; III: strongly poverty related. Figures in parentheses represent 95% uncertainty intervals. For details see statistical appendix.

### R&D expenditure and neglect in R&D

Between 2008 and 2012, for the 26 PRND included in our analysis of neglect, on average a total of US$3,556 million (in nominal 2012 US$) was spent on R&D annually, based on G-FINDER data (23, 24). In the same time period, US$265,920 million (in nominal 2012 US$) was spent in average annually on total health-related R&D worldwide, based on figures published by Chakma et al. (25). Thus, between 2008 and 2012, 1.34% of total global health-related R&D expenditure was spent on the 26 PRND included in our analysis.

**Fig. 2:**
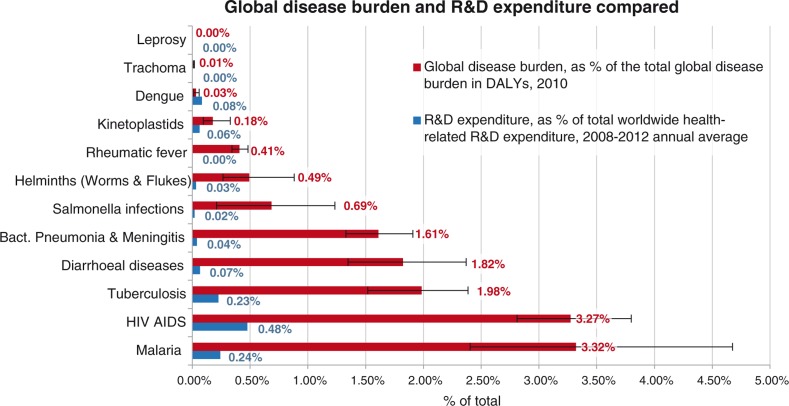
Disease burden in DALYs (as % of total global disease burden, 2010) and R&D Expenditure (as % of total global health R&D Expenditure, annual average for 2008–2010) for 11 diseases and disease groups as defined by G-FINDER. Source: Own calculation based on Global Burden of Disease Study 2010 and G-FINDER data and data published by Chakma et al. DALYs: disability adjusted life years; R&D: research and development.

**Fig. 3:**
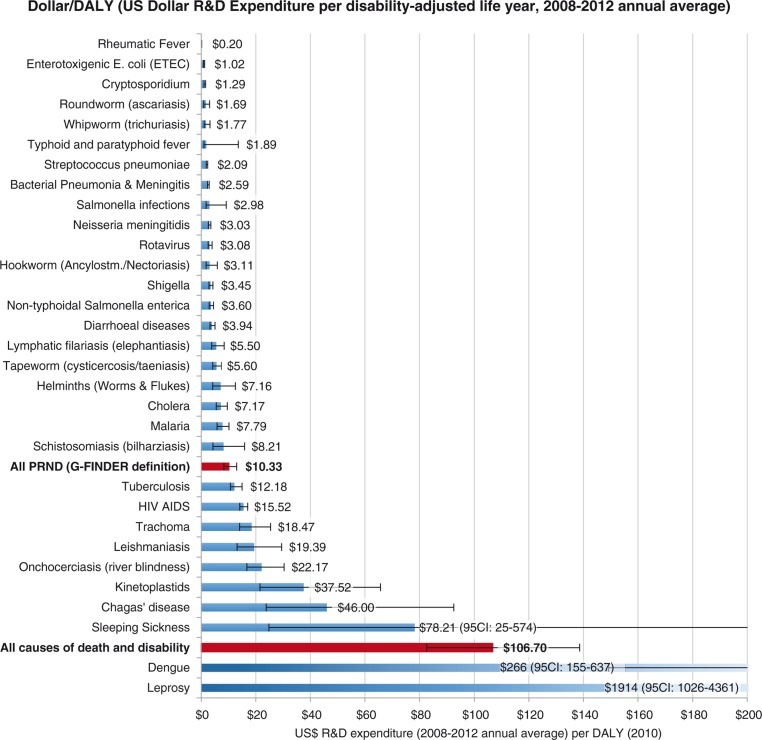
R&D expenditure (2008–2012 annual average, in 2012 nominal US$) per DALY (2010). Source: Own calculations based on Global Burden of Disease Study 2010, G-FINDER data and data published by Chakma et al. DALYs: disability adjusted life years; R&D: research and development.

**Fig. 4:**
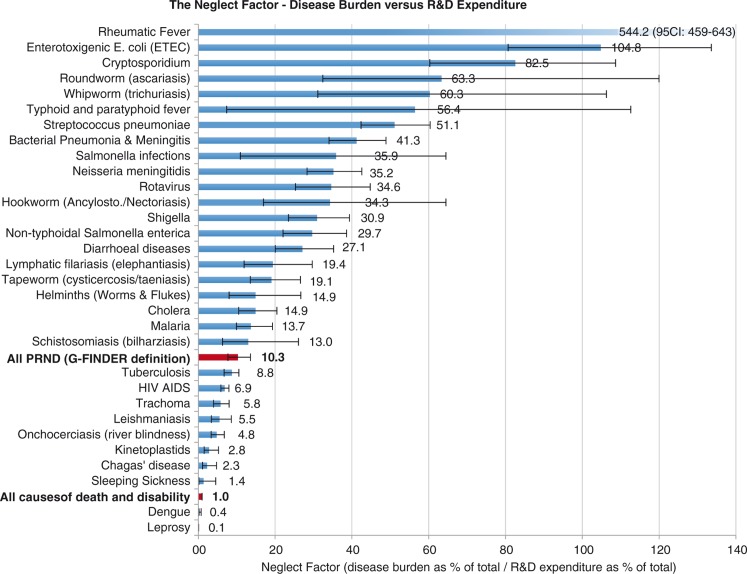
The neglect factor for 26 poverty-related diseases. The neglect factor is the ratio of disease burden in DALYs (as a percentage of the total global disease burden, 2010) versus R&D expenditure (as a percentage of total global health-related R&D expenditure, 2008–2012 average). Source: Own calculations based on Global Burden of Disease Study 2010, G-FINDER data and data published by Chakma et al. DALYs: disability adjusted life years; R&D: research and development.

**Fig. 5:**
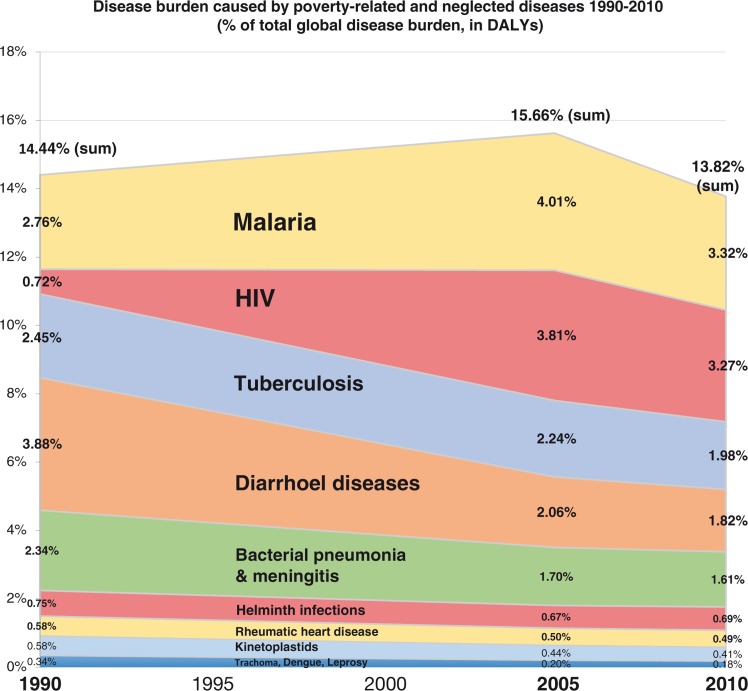
The overall contribution of PRND to the global burden of disease has remained comparatively stable over the past 20 years, with marked changes in the relative weight of individual disease. Source: Own calculations based on Global Burden of Disease Study 2010. Disease groups are defined as in G-FINGER, excluding four diseases for which no disease burden data were available.

**Fig. 6:**
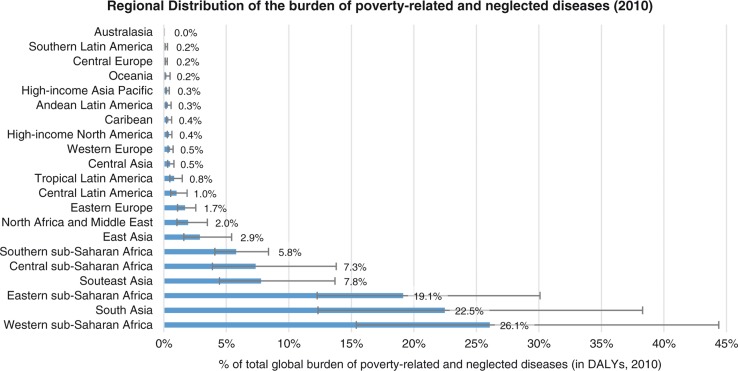
PRND are strongly concentrated in only three world regions: Western and Eastern sub-Saharan Africa, and South Asia together account for more than 60% of the global burden of PRND, illustrating the concentration of PRND in the poorest countries. Source: Own calculations based on Global Burden of Disease Study 2010.

Of 2,490 million DALYs lost in 2010 to all causes of death and disability, 13.8% were caused by the 26 PRND included in our analysis (for details on each of these, see Fig. 2 and Table 4). From 2008 to 2012, on average 107 US$ per DALY was spent on health-related R&D annually. For the 26 PRND in our analysis, only 10.3 US$ per DALY was spent – 10 times less than the global average for all diseases. This is summed up by the NF of 10.3 for these 26 diseases combined, showing that the proportion of the global disease burden caused by these diseases is roughly 10 times larger than the proportion of total global health-related R&D expenditure spent on them. Figures 3 and 4 show detailed results for the 26 diseases and diseases groups. Figure 5 tracks the disease burden caused by these 26 diseases over the past 20 years, and Fig. 6 analyzes the geographical distribution of the disease burden caused by PRND.

**Table 4:** R&D expenditure (2008–2012 annual average) and disease burden (2010) compared

					DALYs per 100,000 inhabitants			
							
Cause or cause group	DALYs (% of total)	R&D (% of total)	Neglect factor	Dollar per DALY	LMIC	HIC	IRF	Disease type
All causes	100 (100–100)	100.00	1 (1–1)	107 (83–139)	38041.42 (27279.01–52476.16)	26421.32 (19916.73–34930.07)	1.44 (.78–2.63)	Ic (Ic-Ic)
All G-FINDER diseases	13.82 (10.35–18.14)	1.34	10.3 (7.7–13.6)	10 (8–13)	5808.1 (4,243–7,765)	468.9 (385–573)	12.4 (7–20)	II (II-II)
HIV AIDS	3.27 (2.81–3.80)	0.48	6.9 (5.9–8.0)	16 (14–17)	1376.3 (1,188–1,571)	108.9 (98–121)	12.6 (10–16)	II (II-II)
Malaria	3.32 (2.40–4.68)	0.24	13.7 (9.9–19.3)	8 (6–10)	1414.6 (961–2,050)	0.1 (0–1)	11465.2 (1,268–1,303,421)	III (III-III)
Tuberculosis	1.98 (1.52–2.39)	0.23	8.8 (6.7–10.6)	12 (11–15)	837.9 (654–1,010)	41.4 (34–51)	20.3 (13–30)	II (II-II)
Diarrheal diseases	1.82 (1.35–2.37)	0.07	27.1 (20.0–35.2)	4 (3–5)	769.0 (533–1,068)	47.3 (31–70)	16.3 (8–35)	II (II-II)
Rotavirus	0.75 (.55–.97)	0.02	34.6 (25.3–44.8)	3 (3–4)	315.8 (221–428)	18.7 (12–27)	16.9 (8–35)	II (II-II)
Enterotoxigenic E. coli (ETEC)	0.28 (.21–.35)	0.00	104.8 (80.7–133.6)	1 (1–1)	114.9 (83–155)	17.5 (11–26)	6.6 (3–14)	II (II-II)
Cholera	0.18 (.13–.25)	0.01	14.9 (10.5–20.5)	7 (6–10)	76.3 (46–119)	0.0 (0–0)	3156.8 (708–13,273)	III (III-III)
Shigella	0.28 (.22–.36)	0.01	30.9 (23.5–39.4)	3 (3–4)	119.3 (87–162)	7.5 (5–11)	15.9 (8–33)	II (II-II)
Cryptosporidium	0.34 (.25–.44)	0.00	82.5 (60.2–108.7)	1 (1–2)	142.6 (95–205)	3.6 (2–6)	39.8 (15–107)	III (II-III)
Dengue	0.03 (.01–.06)	0.08	0.4 (.2–.7)	266 (155–637)	13.3 (5–29)	4.7 (2–8)	2.9 (1–14)	Ic (Ib-II)
Kinetoplastids	0.18 (.10–.33)	0.06	2.8 (1.5–5.3)	38 (22–66)	74.7 (38–140)	5.4 (3–11)	13.9 (3–55)	II (II-III)
Chagas disease	0.02 (.01–.04)	0.01	2.3 (1.1–4.7)	46 (24–93)	8.5 (4–18)	4.9 (2–10)	1.7 (0–8)	Ic (Ib-II)
Leishmaniasis	0.13 (.08–.21)	0.02	5.5 (3.4–8.6)	19 (13–30)	56.7 (33–92)	0.5 (0–1)	117.7 (45–290)	III (III-III)
Sleeping sickness	0.02 (.00–.08)	0.02	1.4 (.2–4.6)	78 (25–574)	9.6 (1–30)	–	a	III (III-III)
Helminths (worms & flukes)	0.49 (.27–.88)	0.03	14.9 (8.0–26.7)	7 (4–13)	209.6 (114–381)	1.6 (1–3)	130.4 (37–479)	III (III-III)
Roundworm (ascariasis)	0.05 (.03–.10)	0.00	63.3 (32.4–119.9)	2 (1–3)	22.4 (12–40)	0.4 (0–1)	50.6 (12–205)	III (II-III)
Hookworm (Ancylostomiasis & Nectoriasis)	0.13 (.06–.24)	0.00	34.3 (17.0–64.5)	3 (2–6)	55.1 (27–102)	0.9 (0–2)	59.5 (16–212)	III (II-III)
Whipworm (trichuriasis)	0.03 (.01–.05)	0.00	60.3 (31.2–106.3)	2 (1–3)	10.9 (6–18)	0.0 (0–0)	1110.4 (323–3,864)	III (III-III)
Lymphatic filariasis (elephantiasis)	0.11 (.07–.17)	0.01	19.4 (11.9–29.7)	5 (4–8)	47.5 (31–69)	–	a	III (III-III)
Onchocerciasis (river blindness)	0.02 (.01–.03)	0.00	4.8 (3.3–6.8)	22 (17–30)	8.5 (6–11)	–	a	III (III-III)
Schistosomiasis (bilharziasis)	0.13 (.06–.27)	0.01	13.0 (6.3–26.1)	8 (4–16)	56.6 (27–127)	–	a	III (III-III)
Tapeworm (cysticercosis/taeniasis)	0.02 (.01–.03)	0.00	19.1 (13.5–26.6)	6 (4–7)	8.6 (6–13)	0.2 (0–0)	37.7 (13–116)	III (II-III)
Bacterial pneumonia & meningitis	1.61 (1.33–1.91)	0.04	41.3 (34.1–48.9)	3 (2–3)	654.9 (528–805)	178.6 (151–209)	3.7 (3–5)	II (Ic-II)
Streptococcus pneumoniae	1.40 (1.16–1.66)	0.03	51.1 (42.4–60.4)	2 (2–2)	567.9 (458–697)	171.0 (145–200)	3.3 (2–5)	II (Ic-II)
Neisseria meningitidis	0.21 (.17–.25)	0.01	35.2 (28.3–42.6)	3 (3–4)	87.0 (70–107)	7.6 (6–9)	11.4 (8–17)	II (II-II)
Salmonella infections	0.69 (.21–1.23)	0.02	35.9 (10.9–64.5)	3 (2–9)	289.7 (83–513)	14.7 (8–23)	19.6 (4–65)	II (II-III)
Non-typhoidal Salmonella enterica (NTS)	0.19 (.14–.25)	0.01	29.7 (22.1–38.6)	4 (3–5)	81.1 (55–117)	10.5 (7–15)	7.7 (4–16)	II (II-II)
Typhoid and paratyphoid fever (S. typhi, S. paratyphi A)	0.49 (.06–.98)	0.01	56.4 (7.4–112.6)	2 (1–14)	208.7 (28–395)	4.2 (1–8)	49.4 (3–725)	III (II-III)
Leprosy	0.00 (.00–.00)	0.00	0.1 (.0–.1)	1,914 (1,026–4,361)	0.1 (0–0)	0.0 (0–0)	65.8 (8–1,110)	III (II-III)
Rheumatic fever	0.41 (.34–.48)	0.00	544.2 (458.6–642.8)	0 (0–0)	162.1 (136–190)	66.2 (58–76)	2.4 (2–3)	Ic (Ic-II)
Trachoma	0.01 (.01–.02)	0.00	5.8 (4.0–8.0)	18 (14–25)	5.7 (4–8)	–	a	III (III-III)

Own calculations based on Global Burden of Disease Study 2010 and G-FINDER data and data published by Chakma et al. (25). DALYs: disability-adjusted life years; LMIC: low- and middle-income countries; HIC: high-income countries; IRF: income relation factor, ratio of DALYs per 100,000 inhabitants in LMIC versus HIC; Disease Type Ia: strongly affluence-related diseases; Ib: moderately affluence related; Ic: unrelated to the level of economic development; II: moderately poverty related; III: strongly poverty related. aDisease not prevalent in HIC.

## Discussion

### Relatedness of diseases, injuries, and risk factors to the level of economic development

Our analysis reveals that the number of poverty-related diseases is considerably larger than existing definitions of PRND suggest. Of the 241 individual diseases and injuries analyzed in the GBD 2010, approximately one third (81) are either strongly or moderately poverty related, causing 38% of the global disease burden in 2010, down from 51% in 1990.

The existing Type I disease category (0≥IRF<3), encompassing all diseases not related to poverty, contains 165 diseases and injuries, responsible for 61% of the global disease burden in 2010. This disease group includes diseases such as Dengue (IRF 2.9), which causes three times more DALY per capita in LMIC compared to HIC, as well as diseases such as prostate cancer (IRF 0.16) causing approximately seven times more DALYs per capita in HIC than in LMIC. The large range of this category, and the large number diseases included, warrant a substratification.

Based on our proposed substratification, 43 diseases and injuries are either strongly affluence related (Type Ia, IRF<0.33) or moderately affluence related (Type Ib, 0.33≥IRF<0.66), causing 13.2% of the global disease burden, a figure which has remained comparatively stable since 1990. In addition, approximately half of all diseases and injuries, responsible for half of the global disease burden (49% in 2010, up from 34% in 1990), are unrelated to the level of economic development (disease Type Ic, 0.66≥IRF<3). Most non-communicable diseases, including many cardiovascular and neuropsychiatric disorders, are found in this category.

This highlights general patterns in the global epidemiological transition. The IRF figures clearly illustrate the double burden of communicable and non-communicable diseases faced by LMIC, showing that while communicable diseases (IRF 10.3) are strongly concentrated in LMIC, non-communicable diseases (IRF 0.8) are causing almost as many DALYs per capita in LMIC as in HIC. Neoplasms (IRF 0.6) are the only main disease group causing a considerably smaller number of DALYs per capita in LMIC than in HIC, while still qualifying as only moderately affluence related.

Remarkably, this double burden has decreased since 1990, as the burden of non-communicable diseases has decreased simultaneously in LMIC and in HIC (IRF 0.8 constant since 1990), while the burden of communicable diseases has decreased more rapidly in LMIC than in HIC (IRF 10.3 in 2010, down from 12.8 in 1990). The burden of injuries has decreased both in LMIC and in HIC, but slightly faster in HIC (IRF 1.7 in 2010, up from 1.6 in 1990). Thus, while it is true that LMIC face actually a triple burden of communicable diseases, non-communicable diseases, and injuries, this phenomenon has grown less acute since 1990.

Our analysis of the relatedness of risk factors to the level of economic development reveals similar patterns for the year 2010, showing a double burden of risk factors in LMIC: among the 10 major risk factor clusters analyzed in the GBD 2010, there are two which are strongly poverty related (unimproved water and sanitation, and child and maternal undernutrition), one which is moderately poverty related (air pollution) and seven which are unrelated to the level of economic development, causing a similar amount of DALYs in HIC and in LMIC. Global disparities in risk factor exposure patterns were higher than those in disease burden patterns in 1990, and have converged more consistently since then.

Among the 81 strongly or moderately poverty-related conditions, a considerable number are infectious diseases which can be treated or prevented with existing pharmaceuticals. For others, for example, accidents, intentional injuries, and certain maternal and neonatal conditions such as birth trauma and abortion, pharmaceuticals are of limited usefulness. This shows that the lack of pharmaceutical R&D on neglected diseases is only one among many health challenges specific to LMIC. Moreover, this highlights that for a considerable number of poverty-related causes of death and disability, policy action beyond the traditional field of health policy might be needed.

### Health-related R&D expenditure and neglect in R&D

The 26 PRND included in our analysis are responsible for 14% of the global disease burden, but receive only 1.3% of global health-related R&D expenditure. In 1990, the Global Commission on Health Research identified a ‘10/90 Gap’, in global health R&D, based on the assumption that in 1990, less than 5% of global health R&D was spent on diseases specific to developing countries, while 93% of the world's preventable mortality occurs in this group of countries (26). Following this commonly used terminology and based on the results of our analysis, we can thus identify a ‘1.3/14 Gap’ in global health-related R&D. However, variation within this group of PRND is large, as shown by the wide range of the NF (median: 19; range: 544 (0.06–544); interquartile range (IQR): 47 (6–52)) and the Dollar per DALY metric (median: 28; range: 9,570 (1–9,571); IQR: 83 (10–94)). Despite this variation, our results clearly show that a large shortfall in R&D funding for PRND persists.

The relative contribution of these 26 PRND to the global disease burden has remained relatively stable over the past 20 years (13.8% in 2010 down from 14.4% in 1990), with shifting shares of individual PRND (see Fig. 5). By contrast, the role of the larger set of strongly and moderately poverty-related diseases identified in the first part of our analysis has decreased strongly from 51% of the global disease burden in 1990 to 38% in 2010. This shows that the 26 PRND in our analysis are, unlike the larger set of poverty-related diseases, not only poverty related but also neglected. While poverty-related diseases more generally are receding, the R&D gap for PRND, and their contribution to the global disease burden, over the past 20 years, has not grown significantly smaller in the course of the global epidemiological transition. It is therefore unlikely to disappear without increased action by public and private actors. For this action to be effective, however, better data on PRND is needed, suggesting a rationale for a WHO Global Health R&D Observatory (13, 17). Moreover, recent national and international policy initiatives such as the WHO R&D Demonstration Projects should be considered in this context (27).

### Limitations

In our categorization of diseases into diseases Types I, II, and III, we followed the approach used by the WHO Secreteriat and Røttingen et al., although noting limitations outlined by them (13, 18). The choice of the cut-off values between the different disease categories is arbitrary to a certain degree, which is aggravated by the fact that the relative size of the disease categories is highly sensitive to the cut-off value. In addition, the DALY figures used are not age weighted.

Moreover, it should be noted that the IRF metric used in our analysis was originally developed for use with data from the GBD 2004 study, which uses a different methodology compared to the one used by GBD 2010. Therefore, our results are not directly comparable to the results reported in the original WHO background document (17) and Røttingen et al. (13). (For details please refer to the methodological annex.) It is also important to note that the IRF captures only health disparities across countries, and not those within countries.

Our analysis of neglect in R&D based on R&D expenditure and disease burden also has a number of limitations. Even for diseases not related to poverty, additional R&D needs specific to resource-poor settings may exist, which may not be addressed by commercially driven R&D geared toward high-income settings. This ‘intra-disease R&D gap’ is not captured by our analysis. Moreover, the disease burden measured in DALYs is a very crude measure for R&D needs. The equitable and efficient level of R&D on a specific disease might depend, among other things, on: 1) the relative epidemiological and public health relevance of the disease; 2) the suitability and viability of existing medical and non-medical prevention and treatment options, and thus the medical need for new or improved pharmaceuticals; 3) the scientific and technical prospects of successfully developing new or improved pharmaceuticals for the disease in question; 4) the potential contribution these new remedies could make in fighting the disease under the given political, economic, social, and socioeconomic conditions. All four factors depend at least partly on facts which are unknown, uncertain, controversial, and/or difficult to assess in a systematic, comprehensive, and scientifically rigorous way, while only the first point is addressed by the DALY measure which we have used. This is a considerable limitation. Particularly important in light of the concentration of PRND in the poorest countries (see Fig. 6) is that weak health systems limit access to existing drugs, and will severely limit the benefits of any new drugs for PRND. This implies that measures to address the R&D gap will only realize their full potential if complemented with a strengthening of national health systems.

Moreover, considerable uncertainties are attached to the data we have used in our analysis, namely the disease burden data and the R&D expenditure data for total global biomedical R&D and for specific neglected diseases, which we have taken from different sources (19, 20, 23, 24). For figures on specific neglected diseases, we used the G-FINDER, which uses very strict inclusion criteria and only considers funding data verified both by donors and receiving R&D organizations (23). This implies that G-FINDER data on R&D funding are lower-bound estimates. By contrast, for total world-wide biomedical R&D expenditure, we used figures provided by Chakma et al., who use publicly reported as well as interpolated data (25). Moreover, uncertainties are attached to the measures used by Chakma et al. for inflation and purchasing power adjustments, as pointed out by Young et al. (28).

For our own calculations, we used the National Institutes of Health Biomedical R&D Price Index to adjust for inflation, which is an equally imperfect measure (28). Uncertainties are also attached to the disease burden data we use (19, 20). These may be particularly large for the neglected tropical diseases we have analyzed (29, 30). Moreover, varying definitions of PRND exist (3, 13, 23, 31–33), of which we have chosen only one.

### Results of similar studies

The results of our study are consistent with earlier studies on disparities in disease burden patterns and global health R&D. Røttingen et al. showed that in 2010 roughly 1% of global health-related R&D investment was spent on PRND (13). Pedrique et al. show that only 1% of clinical trials registered between 1999 and 2011 are devoted to PRND and between 2000 and 2011 only 1.2% of new chemical entities were developed for PRND that accounted for 11% of global disease burden (3, 7). Hotez et al. and Viergever found large variations between selected PRND when estimating neglect in R&D using the Dollar/DALY metric (6, 22). Evans et al. analyze the number of articles, systematic reviews, and clinical trials indexed in MEDLINE for 111 prominent medical conditions, and found that global DALYs for each condition had a small, significant negative relationship with the production of each type of MEDLINE articles for that condition (3). Trouiller et al. had reported earlier that of 1,393 new chemical entities marketed between 1975 and 1999, only 16 or roughly 1% was for tropical diseases and tuberculosis, while these diseases were responsible for 11.4% of the global disease burden (11). However, Cohen et al. found for the same time period and the same set of diseases a considerably higher number of new chemical entities (32 or roughly 2%) (34). Viergever, Karam, and Terry find that for every million DALYs caused by communicable, maternal, perinatal, and nutritional conditions, by noncommunicable diseases, or by injuries, the WHO's International Clinical Trials Registry Platform (ICTRP) database contains an estimated 7.4, 52.4, and 6.0 trials, respectively (35). Despite differences in methodology, scope, and time frame, these findings are consistent with the results of our analysis.

## Conclusions

The disease burden caused by individual diseases, disease groups, and risk factors varies strongly with the level of economic development, as demonstrated by the wide range of the IRF. Communicable, neonatal, maternal, and nutritional disorders cause a 10 times larger disease burden per capita in LMIC than in HIC (IRF 10.3). Non-communicable diseases cause only a slightly smaller disease burden per capita in LMIC than in HIC (IRF 0.8), demonstrating the double burden of communicable and non-communicable diseases in LMIC. The 26 poverty-related diseases included in our analysis of neglect in R&D are responsible for 13.8% of the global disease burden, but receive only 1.34% of global health-related R&D expenditure. These findings reveal a considerable shortfall in R&D funding for PRND. The degree of neglect, however, as captured by the Dollar per DALY metric and the NF, varies considerably among the different PRND.

## Supplementary Material

Poverty-related and neglected diseases – an economic and epidemiological analysis of poverty relatedness and neglect in research and development

Poverty-related and neglected diseases – an economic and epidemiological analysis of poverty relatedness and neglect in research and development

Poverty-related and neglected diseases – an economic and epidemiological analysis of poverty relatedness and neglect in research and development
