# Supplementary material for: Poverty-related and neglected diseases – an economic and epidemiological analysis of poverty relatedness and neglect in research and development
Source: Glob Health Action. 2015 Jan 22;8:10.3402/gha.v8.25818. doi: 10.3402/gha.v8.25818 (PMC4306754; doi:10.3402/gha.v8.25818)
Supplement: Poverty-related and neglected diseases – an economic and epidemiological analysis of poverty relatedness and neglect in research and development [file GHA-8-25818-s001.pdf]

Supplementary file 3 – Full results for all risk factors and risk factor clusters in the GBD 2010

This file is supplementary to: von Philipsborn, Peter; Steinbeis, Fridolin; Bender, Max E.; Regmi, Sadie; Tinnemann, Peter: *Poverty-related and neglected diseases: an economic and epidemiological analysis of poverty relatedness and neglect in research and development.* In: Global Health Action 2014, 7: 25818; http://dx.doi.org/10.3402/gha.v7.25818

Correspondence to: Peter von Philipsborn, Faculty of Medicine, Technische Universität München, Stuntzstraße 12, DE-81667 München, Germany, Email: peter.philipsborn@alumni.lse.ac.uk

This work was supported by the German Research Foundation (DFG) and the Technische Universität München within the funding programme Open Access Publishing.

Relatedness of risk factors and risk factor clusters to the level of economic development

Sources: Own calculations based on Global Burden of Disease Study 2010 data.

Figures in parentheses represent 95% uncertainty intervals.

For details please refer to the methodological annexe (supplementary file 1).

Abbreviations:
DALYs: disability adjusted life years
LMIC: low and middle income countries
HIC: high income countries
IRF: income relation factor, ratio of DALYs per 100,000 inhabitants in LMIC versus HIC
Risk Factor Types:

- Ia: strongly affluence-related
- Ib: moderately-affluence related
- Ic: unrelated to the level of economic development
- II: moderately poverty-related
- III: strongly poverty-related

Risk factor or risk factor cluster	2010				1990			
	DALYs per 100,000 inhabitants		IRF	Risk Factor Type	DALYs per 100,000 inhabitants		IRF	Risk Factor Type
	LMIC	HIC			LMIC	HIC		
**1. Unimproved water and sanitation**	**362 [15-708]**	**3 [0-8]**	**106.8 [2.0-5146.9]**	**III [Ic-III]**	**1191 [61-2175]**	**9 [0-19]**	**137.9 [3.3-6081.3]**	**III [II-III]**
1.1. Unimproved water source	133 [8-281]	2 [0-3]	85.5 [2.4-3097.8]	III [Ic-III]	483 [33-972]	4 [0-9]	121.5 [3.8-4005.0]	III [II-III]
1.2. Unimproved sanitation	255 [6-516]	2 [0-4]	137.4 [1.5-11347.7]	III [Ic-III]	824 [25-1559]	5 [0-11]	168.9 [2.3-13871.5]	III [Ic-III]
**2. Air pollution**	**2649 [2219-3131]**	**590 [497-699]**	**4.5 [3.2-6.3]**	**II [II-II]**	**4776 [3961-5601]**	**1179 [1011-1341]**	**4.0 [3.0-5.5]**	**II [Ic-II]**
2.1. Ambient particulate matter pollution	1203 [1032-1387]	577 [491-668]	2.1 [1.5-2.8]	Ic [Ic-Ic]	1643 [1366-1946]	1112 [973-1248]	1.5 [1.1-2.0]	Ic [Ic-Ic]
2.2. Household air pollution from solid fuels	1848 [1397-2358]	7 [0-27]	279.7 [52.1-74103.8]	III [III-III]	3888 [3038-4705]	79 [26-126]	49.1 [24.1-183.5]	III [II-III]
2.3. Ambient ozone pollution	39 [13-68]	17 [6-32]	2.3 [0.4-12.1]	Ic [Ib-II]	52 [17-95]	28 [10-47]	1.9 [0.4-9.8]	Ic [Ib-II]
**3. Other environmental risks**	**240 [175-323]**	**197 [135-276]**	**1.2 [0.6-2.4]**	**Ic [Ib-Ic]**	**107 [80-139]**	**77 [61-97]**	**1.4 [0.8-2.3]**	**Ic [Ic-Ic]**
3.1. Residential radon	26 [3-72]	58 [8-131]	.5 [0.0-9.5]	Ib [Ia-II]				
3.2. Lead exposure	214 [158-280]	139 [110-174]	1.5 [0.9-2.5]	Ic [Ic-Ic]	107 [80-139]	77 [61-97]	1.4 [0.8-2.3]	Ic [Ic-Ic]
**4. Child and maternal undernutrition**	**2827 [2283-3477]**	**83 [59-118]**	**33.9 [19.3-58.8]**	**II [II-III]**	**7748 [6328-9497]**	**134 [99-176]**	**58.0 [35.9-96.0]**	**III [III-III]**
4.1. Suboptimal breastfeeding	812 [474-1213]	5 [3-8]	160.8 [58.4-475.8]	III [III-III]	2518 [1498-3630]	21 [11-32]	122.6 [47.6-330.3]	III [III-III]
4.1.1. Non-exclusive breastfeeding	703 [378-1065]	4 [2-7]	171,4 [54,0-650,0]	III [III-III]	2200 [1235-3206]	18 [9-28]	125,3 [44,4-374,6]	III [III-III]
4.1.2. Discontinued breastfeeding	110 [10-256]	1 [0-2]	114,9 [4,9-2852,2]	III [II-III]	318 [32-709]	3 [0-6]	106,7 [5,0-2634,1]	III [II-III]
4.2. Childhood underweight	1322 [1040-1674]	3 [2-5]	421.3 [217.2-792.6]	III [III-III]	4520 [3690-5630]	12 [8-17]	362.5 [215.3-682.5]	III [III-III]
4.3. Iron deficiency	813 [564-1147]	70 [47-103]	11.6 [5.5-24.4]	II [II-II]	1167 [836-1620]	91 [66-128]	12.8 [6.5-24.5]	II [II-II]
4.4. Vitamin A deficiency	184 [91-310]	1 [0-3]	149.6 [36.2-716.9]	III [III-III]	692 [321-1287]	3 [1-6]	240.8 [54.7-1268.6]	III [III-III]
4.5. Zinc deficiency	156 [37-301]	4 [2-8]	35.3 [4.5-148.3]	III [II-III]	556 [113-1073]	8 [4-15]	69.2 [7.7-287.7]	III [II-III]
**5. Tobacco smoking, including second-hand smoke**	**2208 [1853-2540]**	**2729 [2376-3072]**	**.8 [0.6-1.1]**	**Ic [Ib-Ic]**	**2722 [2306-3179]**	**3696 [3356-4070]**	**.7 [0.6-0.9]**	**Ic [Ib-Ic]**
5.1. Tobacco smoking	1887 [1546-2204]	2614 [2252-2957]	.7 [0.5-1.0]	Ic [Ib-Ic]	1899 [1572-2288]	3463 [3112-3852]	.5 [0.4-0.7]	Ib [Ib-Ic]
5.2. Second-hand smoke	321 [233-417]	115 [76-161]	2.8 [1.4-5.5]	Ic [Ic-II]	823 [591-1068]	233 [165-300]	3.5 [2.0-6.5]	II [Ic-II]
**6. Alcohol and drug use**	**2201 [1901-2547]**	**2718 [2448-2997]**	**.8 [0.6-1.0]**	**Ic [Ib-Ic]**	**2039 [1765-2371]**	**3376 [3090-3688]**	**.6 [0.5-0.8]**	**Ib [Ib-Ic]**
6.1. Alcohol use	1890 [1630-2199]	2208 [2015-2422]	.9 [0.7-1.1]	Ic [Ic-Ic]	1793 [1555-2094]	2919 [2686-3179]	.6 [0.5-0.8]	Ib [Ib-Ic]

Risk factor or risk factor cluster	2010				1990			
	DALYs per 100,000 inhabitants		IRF	Risk Factor Type	DALYs per 100,000 inhabitants		IRF	Risk Factor Type
	LMIC	HIC			LMIC	HIC		
6.2. Drug use	316 [228-431]	527 [404-674]	.6 [0.3-1.1]	Ib [Ib-Ic]	250 [178-353]	480 [370-617]	.5 [0.3-1.0]	Ib [Ia-Ic]
**7. Physiological risk factors**	**4149 [3766-4519]**	**4764 [4373-5215]**	**.9 [0.7-1.0]**	**Ic [Ic-Ic]**	**3608 [3314-3937]**	**5793 [5426-6145]**	**.6 [0.5-0.7]**	**Ib [Ib-Ic]**
7.1. High fasting plasma glucose	1302 [1030-1592]	1269 [1034-1531]	1.0 [0.7-1.5]	Ic [Ic-Ic]	1029 [828-1244]	1284 [1057-1526]	.8 [0.5-1.2]	Ic [Ib-Ic]
7.2. High total cholesterol	553 [328-792]	843 [651-1059]	.7 [0.3-1.2]	Ib [Ia-Ic]	590 [441-747]	1551 [1310-1809]	.4 [0.2-0.6]	Ib [Ia-Ib]
7.3. High blood pressure	2571 [2213-2915]	2290 [1922-2654]	1.1 [0.8-1.5]	Ic [Ic-Ic]	2410 [2120-2706]	3570 [3235-3897]	.7 [0.5-0.8]	Ic [Ib-Ic]
7.4. High body-mass index	1198 [931-1485]	2317 [1969-2692]	.5 [0.3-0.8]	Ib [Ib-Ic]	756 [565-964]	2089 [1733-2449]	.4 [0.2-0.6]	Ib [Ia-Ib]
7.5. Low bone mineral density	67 [48-86]	130 [95-172]	.5 [0.3-0.9]	Ib [Ia-Ic]	52 [41-67]	95 [71-124]	.5 [0.3-0.9]	Ib [Ia-Ic]
**8. Dietery risk factors and physical inactivity**	**3669 [3347-3977]**	**3924 [3639-4248]**	**.9 [0.8-1.1]**	**Ic [Ic-Ic]**	**3132 [2840-3435]**	**4550 [4194-4859]**	**.7 [0.6-0.8]**	**Ic [Ib-Ic]**
8.1. Diet low in fruits	1582 [1210-1914]	1141 [862-1415]	1.4 [0.9-2.2]	Ic [Ic-Ic]	1521 [1168-1848]	1575 [1168-1946]	1.0 [0.6-1.6]	Ic [Ib-Ic]
8.2. Diet low in vegetables	568 [357-778]	530 [362-693]	1.1 [0.5-2.2]	Ic [Ib-Ic]	564 [350-778]	776 [526-1014]	.7 [0.3-1.5]	Ic [Ib-Ic]
8.3. Diet low in whole grains	621 [481-755]	440 [336-539]	1.4 [0.9-2.2]	Ic [Ic-Ic]	559 [432-685]	559 [425-688]	1.0 [0.6-1.6]	Ic [Ib-Ic]
8.4. Diet low in nuts and seeds	730 [468-956]	846 [538-1101]	.9 [0.4-1.8]	Ic [Ib-Ic]	666 [429-867]	1289 [833-1656]	.5 [0.3-1.0]	Ib [Ia-Ic]
8.5. Diet low in milk	25 [7-43]	62 [18-105]	.4 [0.1-2.4]	Ib [Ia-Ic]	23 [7-38]	60 [18-101]	.4 [0.1-2.2]	Ib [Ia-Ic]
8.6. Diet high in red meat	22 [10-38]	55 [26-86]	.4 [0.1-1.5]	Ib [Ia-Ic]	17 [7-28]	53 [25-83]	.3 [0.1-1.1]	Ia [Ia-Ic]
8.7. Diet high in processed meat	271 [82-483]	499 [182-804]	.5 [0.1-2.6]	Ib [Ia-Ic]	261 [73-465]	669 [201-1123]	.4 [0.1-2.3]	Ib [Ia-Ic]
8.8. Diet high in sugar-sweetened beverages	126 [72-199]	119 [73-182]	1.1 [0.4-2.7]	Ic [Ib-Ic]	101 [52-168]	114 [68-179]	.9 [0.3-2.5]	Ic [Ia-Ic]
8.9. Diet low in fibre	228 [95-371]	306 [149-468]	.7 [0.2-2.5]	Ic [Ia-Ic]	215 [92-347]	444 [204-689]	.5 [0.1-1.7]	Ib [Ia-Ic]
8.10. Diet low in calcium	33 [23-43]	68 [44-94]	.5 [0.3-1.0]	Ib [Ia-Ic]	29 [21-38]	62 [41-85]	.5 [0.2-0.9]	Ib [Ia-Ic]
8.11. Diet low in seafood omega-3 fatty acids	412 [294-528]	406 [291-532]	1.0 [0.6-1.8]	Ic [Ib-Ic]	368 [266-474]	636 [465-807]	.6 [0.3-1.0]	Ib [Ia-Ic]
8.12. Diet low in polyunsaturated fatty acids	163 [75-252]	214 [103-324]	.8 [0.2-2.5]	Ic [Ia-Ic]	159 [75-244]	334 [160-512]	.5 [0.1-1.5]	Ib [Ia-Ic]
8.13. Diet high in trans fatty acids	154 [108-202]	254 [184-330]	.6 [0.3-1.1]	Ib [Ia-Ic]	107 [74-142]	383 [273-492]	.3 [0.2-0.5]	Ia [Ia-Ib]
8.14. Diet high in sodium	907 [583-1212]	806 [512-1080]	1.1 [0.5-2.4]	Ic [Ib-Ic]	844 [542-1125]	1046 [665-1403]	.8 [0.4-1.7]	Ic [Ib-Ic]
8.15. Physical inactivity and low physical activity	958 [793-1134]	1311 [1126-1510]	.7 [0.5-1.0]	Ic [Ib-Ic]	*Not assessed for 1990 because of absence of exposure data*			
**9. Occupational risk factors**	**984 [740-1272]**	**491 [382-623]**	**2.0 [1.2-3.3]**	**Ic [Ic-II]**	**1128 [862-1420]**	**686 [565-831]**	**1.6 [1.0-2.5]**	**Ic [Ic-Ic]**
9.1. Occupational carcinogens	35 [20-52]	62 [44-83]	.6 [0.2-1.2]	Ib [Ia-Ic]	26 [16-41]	68 [51-93]	.4 [0.2-0.8]	Ib [Ia-Ic]
9.1.1. Occupational exposure to asbestos	3 [1-6]	48 [32-66]	,1 [0,0-0,2]	Ia [Ia-Ia]	1 [1-2]	49 [35-69]	,0 [0,0-0,1]	Ia [Ia-Ia]
9.1.2. Occupational exposure to arsenic	1 [0-2]	0 [0-1]	3,1 [0,6-15,1]	II [Ib-II]	1 [0-2]	0 [0-1]	1,8 [0,4-8,6]	Ic [Ib-II]
9.1.3. Occupational exposure to benzene	1 [1-2]	2 [1-3]	,8 [0,2-4,7]	Ic [Ia-II]	1 [0-2]	2 [1-3]	,6 [0,1-3,5]	Ib [Ia-II]
9.1.4. Occupational exposure to beryllium	0 [0-0]	0 [0-0]	2,7 [0,6-11,8]	Ic [Ib-II]	0 [0-0]	0 [0-0]	1,3 [0,3-5,5]	Ic [Ia-II]

Risk factor or risk factor cluster	2010				1990			
	DALYs per 100,000 inhabitants		IRF	Risk Factor Type	DALYs per 100,000 inhabitants		IRF	Risk Factor Type
	LMIC	HIC			LMIC	HIC		
9.1.5. Occupational exposure to cadmium	0 [0-0]	0 [0-0]	2,5 [0,7-9,2]	Ic [Ic-II]	0 [0-0]	0 [0-0]	1,2 [0,3-4,4]	Ic [Ia-II]
9.1.6. Occupational exposure to chromium	1 [0-1]	0 [0-0]	2,5 [1,0-6,0]	Ic [Ic-II]	1 [0-1]	0 [0-1]	1,2 [0,5-3,0]	Ic [Ib-II]
9.1.7 Occupational exposure to diesel engine exhaust	8 [4-13]	3 [2-5]	2,6 [0,9-6,8]	Ic [Ic-II]	7 [4-11]	5 [3-7]	1,5 [0,5-4,0]	Ic [Ib-II]
9.1.8. Occupational exposure to second-hand smoke	9 [6-12]	4 [3-6]	2,0 [1,0-3,8]	Ic [Ic-II]	7 [5-10]	6 [4-8]	1,3 [0,6-2,5]	Ic [Ib-Ic]
9.1.9. Occupational exposure to formaldehyde	0 [0-1]	0 [0-0]	4,9 [1,2-21,5]	II [Ic-II]	0 [0-1]	0 [0-0]	3,7 [0,9-15,5]	II [Ic-II]
9.1.10. Occupational exposure to nickel	3 [1-7]	1 [0-3]	2,7 [0,3-21,4]	Ic [Ib-II]	3 [1-5]	2 [1-4]	1,3 [0,2-10,6]	Ic [Ia-II]
9.1.11. Occupational exposure to polycyclic aromatic hydrocarbons	2 [1-3]	1 [0-1]	2,3 [0,7-8,2]	Ic [Ic-II]	1 [0-2]	1 [0-1]	1,1 [0,3-4,1]	Ic [Ib-II]
9.1.12. Occupational exposure to silica	6 [4-9]	2 [2-3]	2,7 [1,1-5,7]	Ic [Ic-II]	5 [3-7]	3 [2-4]	1,5 [0,7-3,2]	Ic [Ic-II]
9.1.13. Occupational exposure to sulphuric acid	1 [0-2]	1 [0-2]	1,4 [0,2-15,5]	Ic [Ia-II]	1 [0-2]	1 [0-3]	,9 [0,1-10,3]	Ic [Ia-II]
9.2. Occupational asthmagens	31 [20-50]	21 [14-31]	1.5 [0.7-3.7]	Ic [Ib-II]	43 [26-74]	26 [18-38]	1.6 [0.7-4.1]	Ic [Ic-II]
9.3. Occupational particulate matter. gases. and fumes	150 [70-237]	38 [14-66]	4.0 [1.1-16.5]	II [Ic-II]	210 [98-327]	41 [16-70]	5.1 [1.4-20.4]	II [Ic-II]
9.4. Occupational noise	56 [33-90]	19 [11-31]	3.0 [1.1-8.4]	Ic [Ic-II]	60 [35-97]	26 [15-44]	2.3 [0.8-6.4]	Ic [Ic-II]
9.5. Occupational risk factors for injuries	382 [250-574]	112 [93-140]	3.4 [1.8-6.2]	II [Ic-II]	434 [285-629]	260 [220-302]	1.7 [0.9-2.9]	Ic [Ic-Ic]
9.6. Occupational low back pain	330 [214-476]	240 [156-347]	1.4 [0.6-3.1]	Ic [Ib-II]	355 [228-513]	264 [173-376]	1.3 [0.6-3.0]	Ic [Ib-Ic]
**10. Sexual abuse and violence**	**353 [245-482]**	**286 [212-379]**	**1.2 [0.6-2.3]**	**Ic [Ib-Ic]**	*Not assessed for 1990 because of absence of exposure data*			
10.1. Childhood sexual abuse	110 [78-150]	136 [102-176]	.8 [0.4-1.5]	Ic [Ib-Ic]				
10.2. Intimate partner violence	259 [162-380]	161 [101-240]	1.6 [0.7-3.8]	Ic [Ic-II]				
