# Supplementary material for: Poverty-related and neglected diseases – an economic and epidemiological analysis of poverty relatedness and neglect in research and development
Source: Glob Health Action. 2015 Jan 22;8:10.3402/gha.v8.25818. doi: 10.3402/gha.v8.25818 (PMC4306754; doi:10.3402/gha.v8.25818)
Supplement: Poverty-related and neglected diseases – an economic and epidemiological analysis of poverty relatedness and neglect in research and development [file GHA-8-25818-s002.pdf]

Supplementary file 2 – Full results for all causes of death and disability and all cause groups in the GBD 2010

This file is supplementary to: von Philipsborn, Peter; Steinbeis, Fridolin; Bender, Max E.; Regmi, Sadie; Tinnemann, Peter: *Poverty-related and neglected diseases: an economic and epidemiological analysis of poverty relatedness and neglect in research and development.* In: Global Health Action 2014, 7: 25818; http://dx.doi.org/10.3402/gha.v7.25818

Correspondence to: Peter von Philipsborn, Faculty of Medicine, Technische Universität München, Stuntzstraße 12, DE-81667 München, Germany, Email: peter.philipsborn@alumni.lse.ac.uk

This work was supported by the German Research Foundation (DFG) and the Technische Universität München within the funding programme Open Access Publishing.

Relatedness of causes of death and disability and cause groups to the level of economic development

Sources: Own calculations based on Global Burden of Disease Study 2010 data.

Figures in parentheses represent 95% uncertainty intervals.

For details please refer to the methodological annexe (supplementary file 1).

Abbreviations:
DALYs: disability adjusted life years
LMIC: low and middle income countries
HIC: high income countries
IRF: income relation factor, ratio of DALYs per 100,000 inhabitants in LMIC versus HIC
Disease Types:

- Ia: strongly affluence-related
- Ib: moderately-affluence related
- Ic: unrelated to the level of economic development
- II: moderately poverty-related
- III: strongly poverty-related

Cause or cause group	2010				1990			
	DALYs per 100,000 inhabitants		IRF	Disease Type	DALYs per 100,000 inhabitants		IRF	Disease Type
	LMIC	HIC			LMIC	HIC		
**0. All causes**	**38041 [27279-52476]**	**26421 [19917-34930]**	**1.4 [0.8-2.6]**	**Ic [Ic-Ic]**	**51463 [36115-73296]**	**28629 [22174-36789]**	**1.8 [1.0-3.3]**	**Ic [Ic-II]**
**1. Communicable, maternal, neonatal, and nutritional disorders**	**14604 [10322-20676]**	**1416 [1084-1869]**	**10.3 [5.5-19.1]**	**II [II-II]**	**26602 [18241-39060]**	**2084 [1628-2716]**	**12.8 [6.7-24.0]**	**II [II-II]**
**1.1 HIV/AIDS and tuberculosis**	**2214 [1842-2581]**	**150 [132-171]**	**14.7 [10.8-19.6]**	**II [II-II]**	**1746 [1444-2160]**	**343 [305-384]**	**5.1 [3.8-7.1]**	**II [II-II]**
1.1.1 Tuberculosis	*838 [654-1010]*	*41 [34-51]*	*20.3 [12.9-29.9]*	*II [II-II]*	*1383 [1166-1667]*	*90 [75-106]*	*15.3 [11.0-22.1]*	*II [II-II]*
1.1.2 HIV/AIDS	*1376 [1188-1571]*	*109 [98-121]*	*12.6 [9.8-16.0]*	*II [II-II]*	*363 [277-493]*	*253 [230-278]*	*1.4 [1.0-2.1]*	*Ic [Ic-Ic]*
1.1.2.1 HIV disease resulting in mycobacterial infection	255 [222-288]	3 [3-3]	85.3 [64.5-110.3]	III [III-III]	73 [57-99]	8 [7-9]	9.0 [6.1-13.8]	II [II-II]
1.1.2.2 HIV disease resulting in other specified or unspecified diseases	1121 [966-1283]	106 [95-117]	10.6 [8.2-13.4]	II [II-II]	290 [221-394]	245 [223-269]	1.2 [0.8-1.8]	Ic [Ic-Ic]
**1.2 Diarrhoea, lower respiratory infections, meningitis, and other common infectious diseases**	**4720 [3173-7104]**	**695 [534-921]**	**6.8 [3.4-13.3]**	**II [II-II]**	**12252 [8029-19289]**	**840 [644-1139]**	**14.6 [7.0-29.9]**	**II [II-II]**
1.2.1 Diarrhoeal diseases	*1511 [1030-2126]*	*118 [75-178]*	*12.8 [5.8-28.3]*	*II [II-II]*	*4172 [2950-5767]*	*129 [78-203]*	*32.3 [14.5-73.9]*	*II [II-III]*
1.2.1.1 Cholera	76 [46-119]	0 [0-0]	3156.8 [708.5-13273.4]	III [III-III]	224 [147-338]	0 [0-1]	907.2 [259.7-3063.1]	III [III-III]
1.2.1.2 Other salmonella infections	81 [55-117]	11 [7-15]	7.7 [3.8-16.0]	II [II-II]	216 [146-316]	10 [6-14]	22.4 [10.2-48.9]	II [II-III]
1.2.1.3 Shigellosis	119 [87-162]	7 [5-11]	15.9 [7.9-32.7]	II [II-II]	309 [228-414]	8 [5-12]	39.8 [19.1-85.1]	III [II-III]
1.2.1.4 Enteropathogenic E coli infection	127 [78-194]	9 [5-16]	14.3 [5.0-42.6]	II [II-III]	405 [259-596]	13 [7-23]	31.5 [11.4-90.8]	II [II-III]
1.2.1.5 Enterotoxigenic E coli infection	115 [83-155]	18 [11-26]	6.6 [3.2-13.6]	II [II-II]	285 [208-391]	17 [11-26]	16.9 [8.2-37.1]	II [II-III]
1.2.1.6 Campylobacter enteritis	127 [82-185]	11 [7-16]	12.1 [5.2-28.0]	II [II-II]	378 [256-533]	11 [7-18]	32.9 [14.2-77.2]	II [II-III]
1.2.1.7 Amoebiasis	38 [26-53]	4 [2-6]	10.1 [4.4-23.1]	II [II-II]	81 [56-116]	3 [2-5]	28.5 [12.4-69.7]	II [II-III]
1.2.1.8 Cryptosporidiosis	143 [95-205]	4 [2-6]	39.8 [15.1-107.2]	III [II-III]	431 [301-597]	6 [3-10]	73.8 [29.4-196.4]	III [II-III]
1.2.1.9 Rotaviral enteritis	316 [221-428]	19 [12-27]	16.9 [8.1-35.0]	II [II-II]	961 [730-1243]	23 [15-35]	41.1 [20.8-82.9]	III [II-III]
1.2.1.10 Other diarrheal diseases	368 [255-509]	37 [24-55]	9.9 [4.6-21.3]	II [II-II]	881 [619-1223]	38 [23-60]	23.0 [10.3-53.5]	II [II-III]
1.2.2 Typhoid and paratyphoid fevers	*209 [28-395]*	*4 [1-8]*	*49.4 [3.4-725.3]*	*III [II-III]*	*211 [28-397]*	*5 [1-9]*	*44.6 [3.2-666.1]*	*III [II-III]*
1.2.3 Lower respiratory infections	*1893 [1465-2403]*	*450 [379-530]*	*4.2 [2.8-6.3]*	*II [Ic-II]*	*4615 [3510-5938]*	*528 [453-613]*	*8.7 [5.7-13.1]*	*II [II-II]*
1.2.3.1 Influenza	315 [250-391]	84 [71-98]	3.8 [2.6-5.5]	II [Ic-II]	722 [555-914]	98 [85-112]	7.4 [4.9-10.8]	II [II-II]

Cause or cause group	2010				1990			
	DALYs per 100,000 inhabitants		IRF	Disease Type	DALYs per 100,000 inhabitants		IRF	Disease Type
	LMIC	HIC			LMIC	HIC		
1.2.3.2 Pneumococcal pneumonia	432 [348-529]	165 [140-193]	2.6 [1.8-3.8]	Ic [Ic-II]	954 [759-1189]	187 [163-215]	5.1 [3.5-7.3]	II [II-II]
1.2.3.3 H influenzae type B pneumonia	358 [276-455]	40 [34-47]	8.9 [5.8-13.4]	II [II-II]	993 [765-1252]	56 [48-65]	17.7 [11.8-26.0]	II [II-II]
1.2.3.4 Respiratory syncytial virus pneumonia	349 [250-466]	9 [7-11]	38.9 [22.5-65.3]	III [II-III]	1024 [754-1341]	21 [16-28]	47.9 [27.1-82.9]	III [II-III]
1.2.3.5 Other lower respiratory infections	441 [340-562]	151 [126-181]	2.9 [1.9-4.5]	Ic [Ic-II]	922 [677-1242]	167 [141-194]	5.5 [3.5-8.8]	II [II-II]
1.2.4 Upper respiratory infections	*27 [15-48]*	*26 [12-48]*	*1.1 [0.3-4.0]*	*Ic [Ia-II]*	*33 [19-55]*	*30 [14-56]*	*1.1 [0.3-3.9]*	*Ic [Ib-II]*
1.2.5 Otitis media	*72 [43-127]*	*47 [30-71]*	*1.5 [0.6-4.2]*	*Ic [Ib-II]*	*84 [48-180]*	*54 [35-82]*	*1.6 [0.6-5.2]*	*Ic [Ib-II]*
1.2.6 Meningitis	*496 [392-628]*	*36 [29-44]*	*13.9 [8.9-21.4]*	*II [II-II]*	*851 [655-1128]*	*64 [53-77]*	*13.3 [8.5-21.1]*	*II [II-II]*
1.2.6.1 Pneumococcal meningitis	136 [110-168]	6 [5-7]	24.0 [15.7-36.3]	II [II-III]	214 [169-277]	11 [9-14]	19.2 [12.4-30.6]	II [II-II]
1.2.6.2 H influenzae type B meningitis	113 [86-149]	3 [3-4]	33.8 [20.7-55.9]	II [II-III]	230 [172-314]	9 [7-11]	25.9 [15.4-43.7]	II [II-III]
1.2.6.3 Meningococcal infection	87 [70-107]	8 [6-9]	11.4 [7.5-17.1]	II [II-II]	130 [102-170]	14 [12-16]	9.4 [6.2-14.6]	II [II-II]
1.2.6.4 Other meningitis	160 [126-203]	19 [16-24]	8.4 [5.4-12.9]	II [II-II]	278 [212-367]	30 [26-35]	9.3 [6.0-14.4]	II [II-II]
1.2.7 Encephalitis	*121 [101-145]*	*5 [4-5]*	*26.9 [19.1-38.3]*	*II [II-III]*	*231 [191-285]*	*7 [6-8]*	*32.1 [23.5-45.3]*	*II [II-III]*
1.2.8 Diphtheria	*4 [0-35]*	*0 [0-1]*	*40.8*	*III [Ia-III]*	*12 [0-99]*	*0 [0-2]*	*44.2*	*III [Ia-III]*
1.2.9 Whooping cough	*119 [2-578]*	*4 [0-17]*	*31.2 [0.1-2578.2]*	*II [Ia-III]*	*325 [5-1565]*	*14 [1-63]*	*23.8 [0.1-2238.0]*	*II [Ia-III]*
1.2.10 Tetanus	*80 [38-155]*	*0 [0-1]*	*431.9 [55.1-7525.6]*	*III [III-III]*	*499 [286-826]*	*1 [0-3]*	*538.6 [84.5-5626.7]*	*III [III-III]*
1.2.11 Measles	*178 [56-419]*	*1 [0-2]*	*296.4 [36.4-1594.9]*	*III [III-III]*	*1202 [336-2943]*	*2 [1-4]*	*512.1 [84.1-2682.3]*	*III [III-III]*
1.2.12 Varicella	*9 [2-45]*	*6 [3-15]*	*1.5 [0.1-16.3]*	*Ic [Ia-II]*	*18 [2-107]*	*6 [3-18]*	*3.0 [0.1-42.9]*	*II [Ia-III]*
**1.3 Neglected tropical diseases and malaria**	**1856 [1200-2863]**	**25 [13-45]**	**74.8 [26.4-224.2]**	**III [II-III]**	**2367 [1507-3760]**	**38 [19-69]**	**62.8 [21.9-194.3]**	**III [II-III]**
1.3.1 Malaria	*1415 [961-2050]*	*0 [0-1]*	*11465.2 [1267.7-1303421.3]*	*III [III-III]*	*1581 [1111-2214]*	*0 [0-2]*	*3706.0 [452.7-254847.1]*	*III [III-III]*
1.3.2 Chagas disease	*8 [4-18]*	*5 [2-10]*	*1.7 [0.4-8.1]*	*Ic [Ib-II]*	*12 [6-21]*	*7 [3-13]*	*1.6 [0.5-6.2]*	*Ic [Ib-II]*
1.3.3 Leishmaniasis	*57 [33-92]*	*0 [0-1]*	*117.7 [45.3-289.9]*	*III [III-III]*	*134 [71-238]*	*1 [0-1]*	*194.6 [67.7-528.0]*	*III [III-III]*
1.3.4 African trypanosomiasis	*10 [1-30]*	*0 [0-0]*	*-*	*III [III-III]*	*47 [7-115]*	*0*	*-*	*III [III-III]*
1.3.5 Schistosomiasis	*57 [27-127]*	*0 [0-0]*	*-*	*III [III-III]*	*49 [22-120]*	*0*	*-*	*III [III-III]*
1.3.6 Cysticercosis	*9 [6-13]*	*0 [0-0]*	*37.7 [13.2-116.3]*	*III [II-III]*	*12 [8-17]*	*1 [0-1]*	*19.3 [7.9-48.0]*	*II [II-III]*
1.3.7 Echinococcosis	*2 [1-6]*	*1 [1-3]*	*1.6 [0.2-12.2]*	*Ic [Ia-II]*	*3 [1-9]*	*2 [1-4]*	*1.8 [0.2-16.0]*	*Ic [Ia-II]*
1.3.8 Lymphatic filariasis	*47 [31-69]*	*0 [0-0]*	*-*	*III [III-III]*	*54 [35-77]*	*2 [1-3]*	*26.9 [11.9-59.1]*	*II [II-III]*
1.3.9 Onchocerciasis	*8 [6-11]*	*0 [0-0]*	*-*	*III [III-III]*	*12 [8-16]*	*0 [0-0]*	*-*	*III [III-III]*
1.3.10 Trachoma	*6 [4-8]*	*0 [0-0]*	*-*	*III [III-III]*	*3 [2-5]*	*0 [0-0]*	*-*	*III [III-III]*

Cause or cause group	2010				1990			
	DALYs per 100,000 inhabitants		IRF	Disease Type	DALYs per 100,000 inhabitants		IRF	Disease Type
	LMIC	HIC			LMIC	HIC		
1.3.11 Dengue	*13 [5-29]*	*5 [2-8]*	*2.9 [0.6-14.0]*	*Ic [Ib-II]*	*15 [4-41]*	*6 [2-10]*	*2.6 [0.4-18.3]*	*Ic [Ib-II]*
1.3.12 Yellow fever	*0 [0-0]*	*0 [0-0]*	*-*	*III [III-III]*	*0 [0-0]*	*0 [0-0]*	*-*	*III [III-III]*
1.3.13 Rabies	*25 [11-51]*	*0 [0-1]*	*51.4 [13.1-277.4]*	*III [II-III]*	*74 [34-155]*	*1 [0-2]*	*84.7 [21.4-312.2]*	*III [II-III]*
1.3.14 Intestinal nematode infections	*88 [45-161]*	*1 [1-3]*	*64.1 [16.7-235.0]*	*III [II-III]*	*205 [107-355]*	*3 [2-6]*	*64.4 [18.8-216.1]*	*III [II-III]*
1.3.14.1 Ascariasis	22 [12-40]	0 [0-1]	50.6 [12.1-204.7]	III [II-III]	96 [52-164]	2 [1-4]	44.3 [13.4-145.7]	III [II-III]
1.3.14.2 Trichuriasis	11 [6-18]	0 [0-0]	1110.4 [322.7-3864.4]	III [III-III]	20 [11-33]	0 [0-0]	289.9 [85.4-999.7]	III [III-III]
1.3.14.3 Hookworm disease	55 [27-102]	1 [0-2]	59.5 [16.0-211.9]	III [II-III]	90 [45-158]	1 [0-2]	94.2 [25.9-327.4]	III [II-III]
1.3.15 Food-borne trematodiases	*32 [11-85]*	*3 [1-6]*	*10.2 [1.7-60.2]*	*II [Ic-III]*	*54 [12-198]*	*6 [2-13]*	*9.6 [0.9-90.1]*	*II [Ic-III]*
1.3.16 Other neglected tropical diseases	*79 [57-112]*	*8 [5-12]*	*9.8 [4.8-21.2]*	*II [II-II]*	*113 [79-179]*	*10 [7-14]*	*11.7 [5.6-26.6]*	*II [II-II]*
**1.4 Maternal disorders**	**273 [206-365]**	**14 [8-24]**	**19.9 [8.5-44.9]**	**II [II-III]**	**490 [385-627]**	**16 [12-23]**	**30.2 [16.7-50.7]**	**II [II-III]**
1.4.1 Maternal hemorrhage	*56 [44-68]*	*2 [1-2]*	*35.2 [20.0-60.3]*	*III [II-III]*	*109 [87-134]*	*3 [2-4]*	*42.2 [24.8-67.3]*	*III [II-III]*
1.4.2 Maternal sepsis	*22 [17-28]*	*0 [0-1]*	*53.2 [30.5-103.8]*	*III [II-III]*	*47 [37-58]*	*1 [0-1]*	*72.1 [42.9-118.7]*	*III [III-III]*
1.4.3 Hypertensive disorders of pregnancy	*48 [37-60]*	*2 [1-2]*	*31.3 [18.8-56.6]*	*II [II-III]*	*93 [75-116]*	*2 [2-3]*	*39.6 [24.3-62.0]*	*III [II-III]*
1.4.4 Obstructed labor	*30 [16-64]*	*2 [0-8]*	*13.7 [2.1-157.2]*	*II [Ic-III]*	*43 [28-74]*	*1 [0-3]*	*51.4 [10.8-379.3]*	*III [II-III]*
1.4.5 Abortion	*36 [28-46]*	*2 [1-2]*	*20.2 [11.2-38.6]*	*II [II-III]*	*73 [57-92]*	*3 [2-4]*	*26.6 [15.1-43.2]*	*II [II-III]*
1.4.6 Other maternal disorders	*81 [63-99]*	*6 [4-9]*	*13.1 [6.9-24.3]*	*II [II-II]*	*125 [100-153]*	*7 [6-9]*	*17.7 [10.8-26.9]*	*II [II-II]*
**1.5 Neonatal disorders**	**3394 [2455-4644]**	**354 [283-443]**	**9.6 [5.5-16.4]**	**II [II-II]**	**6134 [4350-8216]**	**624 [493-773]**	**9.8 [5.6-16.7]**	**II [II-II]**
1.5.1 Preterm birth complications	*1280 [1019-1564]*	*215 [181-255]*	*6.0 [4.0-8.6]*	*II [II-II]*	*2347 [1834-2842]*	*380 [313-448]*	*6.2 [4.1-9.1]*	*II [II-II]*
1.5.2 Neonatal encephalopathy birth asphyxia and birth trauma	*844 [635-1090]*	*78 [63-97]*	*10.8 [6.5-17.2]*	*II [II-II]*	*1359 [1037-1788]*	*134 [107-165]*	*10.1 [6.3-16.7]*	*II [II-II]*
1.5.3 Sepsis and other infectious disorders of the newborn baby	*753 [443-1238]*	*24 [14-42]*	*30.9 [10.4-89.3]*	*II [II-III]*	*1045 [557-1668]*	*38 [20-63]*	*27.5 [8.9-82.3]*	*II [II-III]*
1.5.4 Other neonatal disorders	*517 [359-751]*	*36 [25-48]*	*14.4 [7.5-30.3]*	*II [II-II]*	*1383 [923-1918]*	*72 [53-97]*	*19.3 [9.5-35.9]*	*II [II-III]*
**1.6 Nutritional deficiencies**	**1440 [1013-1997]**	**118 [78-173]**	**12.2 [5.9-25.5]**	**II [II-II]**	**2527 [1869-3396]**	**148 [104-209]**	**17.0 [8.9-32.5]**	**II [II-II]**
1.6.1 Protein-energy malnutrition	*593 [442-775]*	*19 [13-23]*	*32.0 [19.6-57.6]*	*II [II-III]*	*1380 [1081-1752]*	*24 [18-29]*	*56.9 [36.8-95.6]*	*III [III-III]*
1.6.2 Iodine deficiency	*64 [40-101]*	*28 [17-46]*	*2.3 [0.9-6.0]*	*Ic [Ic-II]*	*69 [44-108]*	*30 [18-49]*	*2.3 [0.9-6.0]*	*Ic [Ic-II]*
1.6.3 Vitamin A deficiency	*14 [10-19]*	*0 [0-0]*	*-*	*III [III-III]*	*17 [12-23]*	*0 [0-0]*	*-*	*III [III-III]*
1.6.4 Iron-deficiency anemia	*764 [518-1097]*	*70 [47-103]*	*11.0 [5.0-23.4]*	*II [II-II]*	*1052 [724-1501]*	*91 [66-127]*	*11.5 [5.7-22.8]*	*II [II-II]*
1.6.5 Other nutritional deficiencies	*5 [3-6]*	*1 [1-2]*	*3.7 [2.2-6.5]*	*II [Ic-II]*	*9 [8-12]*	*3 [2-4]*	*3.1 [2.1-5.4]*	*II [Ic-II]*
**1.7 Other communicable, maternal, neonatal, and nutritional disorders**	**707 [433-1122]**	**61 [36-92]**	**11.6 [4.7-31.0]**	**II [II-II]**	**1087 [657-1612]**	**75 [49-118]**	**14.5 [5.6-33.0]**	**II [II-II]**

Cause or cause group	2010				1990			
	DALYs per 100,000 inhabitants		IRF	Disease Type	DALYs per 100,000 inhabitants		IRF	Disease Type
	LMIC	HIC			LMIC	HIC		
1.7.1 Sexually transmitted diseases excluding HIV	*185 [101-308]*	*16 [7-33]*	*11.4 [3.0-46.1]*	*II [II-III]*	*414 [236-668]*	*24 [11-49]*	*17.0 [4.8-63.3]*	*II [II-III]*
1.7.1.1 Syphilis	163 [93-264]	3 [2-5]	51.8 [17.7-162.5]	III [II-III]	388 [226-616]	6 [4-11]	61.3 [21.4-172.1]	III [II-III]
1.7.1.2 Sexually transmitted chlamydial diseases	11 [4-22]	5 [2-11]	2.1 [0.4-10.0]	Ic [Ib-II]	13 [5-25]	7 [3-14]	1.9 [0.4-8.4]	Ic [Ib-II]
1.7.1.3 Gonococcal infection	4 [2-8]	3 [1-7]	1.3 [0.3-5.9]	Ic [Ia-II]	4 [2-8]	4 [2-8]	1.1 [0.3-4.4]	Ic [Ia-II]
1.7.1.4 Trichomoniasis	3 [0-7]	2 [0-6]	1.2 [0.0-.]	Ic [Ia-III]	3 [0-10]	3 [0-10]	1.0 [0.0-.]	Ic [Ia-III]
1.7.1.5 Other sexually transmitted diseases	4 [2-6]	2 [1-4]	1.6 [0.6-4.1]	Ic [Ib-II]	5 [3-8]	4 [2-7]	1.4 [0.5-3.8]	Ic [Ib-II]
1.7.2 Hepatitis	*225 [119-418]*	*13 [9-19]*	*17.6 [6.3-48.7]*	*II [II-III]*	*235 [129-364]*	*19 [13-27]*	*12.2 [4.8-29.0]*	*II [II-II]*
1.7.2.1 Acute hepatitis A	74 [36-161]	3 [2-4]	29.1 [9.6-98.5]	II [II-III]	112 [57-180]	4 [3-6]	28.2 [10.0-67.3]	II [II-III]
1.7.2.2 Acute hepatitis B	79 [51-114]	7 [5-10]	11.4 [5.2-22.0]	II [II-II]	64 [39-89]	10 [7-13]	6.5 [3.1-12.2]	II [II-II]
1.7.2.3 Acute hepatitis C	8 [5-12]	3 [2-5]	2.5 [1.0-7.0]	Ic [Ic-II]	5 [3-9]	5 [3-9]	1.0 [0.3-3.3]	Ic [Ia-II]
1.7.2.4 Acute hepatitis E	64 [26-131]	0 [0-0]	-	III [III-III]	54 [30-86]	0 [0-0]	-	III [III-III]
1.7.3 Leprosy	*0 [0-0]*	*0 [0-0]*	*65.8 [7.6-1110.3]*	*III [II-III]*	*1 [0-1]*	*0 [0-0]*	*45.9 [7.4-278.4]*	*III [II-III]*
1.7.4 Other infectious diseases	*297 [212-396]*	*32 [21-40]*	*9.2 [5.3-18.9]*	*II [II-II]*	*437 [292-578]*	*31 [26-42]*	*14.0 [7.0-22.5]*	*II [II-II]*
**2. Non-communicable diseases**	**19079 [13870-25683]**	**22470 [16895-29815]**	**.8 [0.5-1.5]**	**Ic [Ib-Ic]**	**19841 [14360-27282]**	**23442 [18081-30168]**	**.8 [0.5-1.5]**	**Ic [Ib-Ic]**
**2.1 Neoplasms**	**2461 [1783-3157]**	**4392 [3401-5596]**	**.6 [0.3-0.9]**	**Ib [Ia-Ic]**	**2459 [1827-3192]**	**4575 [3636-5773]**	**.5 [0.3-0.9]**	**Ib [Ia-Ic]**
2.1.1 Esophageal cancer	*132 [92-168]*	*118 [89-151]*	*1.1 [0.6-1.9]*	*Ic [Ib-Ic]*	*161 [126-212]*	*124 [99-156]*	*1.3 [0.8-2.1]*	*Ic [Ic-Ic]*
2.1.2 Stomach cancer	*235 [169-307]*	*261 [205-356]*	*.9 [0.5-1.5]*	*Ic [Ib-Ic]*	*340 [254-451]*	*403 [317-532]*	*.8 [0.5-1.4]*	*Ic [Ib-Ic]*
2.1.3 Liver cancer	*289 [231-367]*	*219 [176-276]*	*1.3 [0.8-2.1]*	*Ic [Ic-Ic]*	*260 [206-314]*	*204 [160-234]*	*1.3 [0.9-2.0]*	*Ic [Ic-Ic]*
2.1.3.1 Liver cancer secondary to hepatitis B	142 [116-181]	64 [52-81]	2.2 [1.4-3.5]	Ic [Ic-II]	128 [103-153]	61 [47-70]	2.1 [1.5-3.3]	Ic [Ic-II]
2.1.3.2 Liver cancer secondary to hepatitis C	57 [45-71]	78 [63-100]	.7 [0.5-1.1]	Ic [Ib-Ic]	46 [36-55]	72 [57-82]	.6 [0.4-1.0]	Ib [Ib-Ic]
2.1.3.3 Liver cancer secondary to alcohol use	55 [44-71]	53 [42-65]	1.0 [0.7-1.7]	Ic [Ic-Ic]	51 [40-62]	47 [37-54]	1.1 [0.7-1.7]	Ic [Ic-Ic]
2.1.3.4 Other liver cancer	34 [26-45]	24 [18-31]	1.4 [0.9-2.4]	Ic [Ic-Ic]	35 [27-45]	24 [19-28]	1.5 [1.0-2.4]	Ic [Ic-Ic]
2.1.4 Larynx cancer	*34 [17-57]*	*38 [22-66]*	*.9 [0.3-2.6]*	*Ic [Ia-Ic]*	*36 [19-61]*	*54 [30-89]*	*.7 [0.2-2.0]*	*Ic [Ia-Ic]*
2.1.5 Trachea, bronchus, and lung cancers	*397 [287-483]*	*904 [700-1082]*	*.4 [0.3-0.7]*	*Ib [Ia-Ic]*	*349 [266-453]*	*968 [788-1200]*	*.4 [0.2-0.6]*	*Ib [Ia-Ib]*
2.1.6 Breast cancer	*144 [131-158]*	*357 [327-389]*	*.4 [0.3-0.5]*	*Ib [Ib-Ib]*	*120 [111-130]*	*407 [383-438]*	*.3 [0.3-0.3]*	*Ia [Ia-Ib]*
2.1.7 Cervical cancer	*99 [59-142]*	*62 [43-95]*	*1.6 [0.6-3.3]*	*Ic [Ib-II]*	*111 [67-159]*	*82 [54-116]*	*1.3 [0.6-3.0]*	*Ic [Ib-Ic]*
2.1.8 Uterine cancer	*15 [7-23]*	*38 [20-55]*	*.4 [0.1-1.1]*	*Ib [Ia-Ic]*	*15 [9-26]*	*40 [23-67]*	*.4 [0.1-1.1]*	*Ib [Ia-Ic]*
2.1.9 Prostate cancer	*31 [17-47]*	*192 [118-294]*	*.2 [0.1-0.4]*	*Ia [Ia-Ib]*	*21 [12-32]*	*164 [100-242]*	*.1 [0.1-0.3]*	*Ia [Ia-Ia]*
2.1.10 Colon and rectum cancers	*163 [139-194]*	*480 [424-572]*	*.3 [0.2-0.5]*	*Ib [Ia-Ib]*	*147 [119-165]*	*475 [412-536]*	*.3 [0.2-0.4]*	*Ia [Ia-Ib]*
2.1.11 Mouth cancer	*46 [37-54]*	*55 [47-64]*	*.8 [0.6-1.1]*	*Ic [Ib-Ic]*	*39 [31-45]*	*58 [50-64]*	*.7 [0.5-0.9]*	*Ic [Ib-Ic]*
2.1.12 Nasopharynx cancer	*32 [20-45]*	*12 [8-17]*	*2.7 [1.2-5.5]*	*Ic [Ic-II]*	*31 [20-45]*	*13 [9-17]*	*2.5 [1.2-5.2]*	*Ic [Ic-II]*

Cause or cause group	2010				1990			
	DALYs per 100,000 inhabitants		IRF	Disease Type	DALYs per 100,000 inhabitants		IRF	Disease Type
	LMIC	HIC			LMIC	HIC		
2.1.13 Cancer of other part of pharynx and oropharynx	*40 [23-53]*	*39 [24-53]*	*1.0 [0.4-2.2]*	*Ic [Ib-Ic]*	*41 [23-55]*	*34 [22-49]*	*1.2 [0.5-2.5]*	*Ic [Ib-Ic]*
2.1.14 Gallbladder and biliary tract cancer	*38 [22-60]*	*78 [55-113]*	*.5 [0.2-1.1]*	*Ib [Ia-Ic]*	*31 [20-48]*	*77 [53-109]*	*.4 [0.2-0.9]*	*Ib [Ia-Ic]*
2.1.15 Pancreatic cancer	*63 [47-81]*	*243 [181-311]*	*.3 [0.2-0.4]*	*Ia [Ia-Ib]*	*54 [40-71]*	*206 [160-271]*	*.3 [0.1-0.4]*	*Ia [Ia-Ib]*
2.1.16 Malignant melanoma of skin	*10 [6-15]*	*59 [37-88]*	*.2 [0.1-0.4]*	*Ia [Ia-Ib]*	*8 [5-12]*	*54 [36-84]*	*.2 [0.1-0.3]*	*Ia [Ia-Ia]*
2.1.17 Non-melanoma skin cancer	*8 [4-12]*	*35 [26-46]*	*.2 [0.1-0.5]*	*Ia [Ia-Ib]*	*7 [4-11]*	*24 [17-32]*	*.3 [0.1-0.7]*	*Ia [Ia-Ib]*
2.1.18 Ovarian cancer	*52 [35-68]*	*107 [76-143]*	*.5 [0.2-0.9]*	*Ib [Ia-Ic]*	*45 [30-59]*	*113 [84-147]*	*.4 [0.2-0.7]*	*Ib [Ia-Ic]*
2.1.19 Testicular cancer	*4 [2-6]*	*8 [5-12]*	*.5 [0.2-1.2]*	*Ib [Ia-Ic]*	*5 [2-7]*	*10 [6-14]*	*.5 [0.2-1.0]*	*Ib [Ia-Ic]*
2.1.20 Kidney and other urinary organ cancers	*39 [27-51]*	*138 [104-208]*	*.3 [0.1-0.5]*	*Ia [Ia-Ib]*	*30 [20-42]*	*93 [68-123]*	*.3 [0.2-0.6]*	*Ia [Ia-Ib]*
2.1.21 Bladder cancer	*35 [27-42]*	*95 [74-113]*	*.4 [0.2-0.6]*	*Ib [Ia-Ib]*	*34 [26-41]*	*102 [85-126]*	*.3 [0.2-0.5]*	*Ib [Ia-Ib]*
2.1.22 Brain and nervous system cancers	*78 [44-107]*	*146 [89-193]*	*.5 [0.2-1.2]*	*Ib [Ia-Ic]*	*75 [47-110]*	*148 [103-210]*	*.5 [0.2-1.1]*	*Ib [Ia-Ic]*
2.1.23 Thyroid cancer	*12 [8-15]*	*16 [12-20]*	*.7 [0.4-1.3]*	*Ic [Ib-Ic]*	*10 [7-13]*	*15 [11-19]*	*.7 [0.4-1.2]*	*Ic [Ib-Ic]*
2.1.24 Hodgkin's disease	*8 [5-13]*	*15 [10-23]*	*.6 [0.2-1.2]*	*Ib [Ia-Ic]*	*13 [8-19]*	*23 [15-33]*	*.5 [0.2-1.3]*	*Ib [Ia-Ic]*
2.1.25 Non-Hodgkin lymphoma	*76 [56-92]*	*140 [114-160]*	*.5 [0.4-0.8]*	*Ib [Ib-Ic]*	*75 [54-96]*	*140 [121-161]*	*.5 [0.3-0.8]*	*Ib [Ib-Ic]*
2.1.26 Multiple myeloma	*14 [8-19]*	*67 [43-95]*	*.2 [0.1-0.4]*	*Ia [Ia-Ib]*	*11 [7-16]*	*61 [43-89]*	*.2 [0.1-0.4]*	*Ia [Ia-Ib]*
2.1.27 Leukemia	*134 [103-162]*	*167 [136-203]*	*.8 [0.5-1.2]*	*Ic [Ib-Ic]*	*166 [128-208]*	*191 [155-235]*	*.9 [0.5-1.3]*	*Ic [Ib-Ic]*
2.1.28 Other neoplasms	*231 [157-315]*	*304 [237-397]*	*.8 [0.4-1.3]*	*Ic [Ib-Ic]*	*223 [163-289]*	*294 [233-382]*	*.8 [0.4-1.2]*	*Ic [Ib-Ic]*
**2.2 Cardiovascular and circulatory diseases**	**4301 [3639-4986]**	**4290 [3877-4973]**	**1.0 [0.7-1.3]**	**Ic [Ic-Ic]**	**4334 [3670-5161]**	**5774 [5259-6239]**	**.8 [0.6-1.0]**	**Ic [Ib-Ic]**
2.2.1 Rheumatic heart disease	*162 [136-190]*	*66 [58-76]*	*2.4 [1.8-3.3]*	*Ic [Ic-II]*	*304 [265-355]*	*129 [115-142]*	*2.3 [1.9-3.1]*	*Ic [Ic-II]*
2.2.2 Ischemic heart disease	*1847 [1642-2014]*	*2147 [2009-2420]*	*.9 [0.7-1.0]*	*Ic [Ic-Ic]*	*1660 [1549-1846]*	*3146 [2940-3317]*	*.5 [0.5-0.6]*	*Ib [Ib-Ib]*
2.2.3 Cerebrovascular disease	*1556 [1282-1861]*	*1109 [1000-1293]*	*1.4 [1.0-1.9]*	*Ic [Ic-Ic]*	*1659 [1321-2033]*	*1522 [1365-1642]*	*1.1 [0.8-1.5]*	*Ic [Ic-Ic]*
2.2.3.1 Ischemic stroke	562 [471-678]	641 [583-763]	.9 [0.6-1.2]	Ic [Ib-Ic]	564 [466-700]	841 [755-890]	.7 [0.5-0.9]	Ic [Ib-Ic]
2.2.3.2 Hemorrhagic and other non-ischemic stroke	994 [811-1183]	468 [417-531]	2.1 [1.5-2.8]	Ic [Ic-Ic]	1094 [855-1333]	681 [609-752]	1.6 [1.1-2.2]	Ic [Ic-Ic]
2.2.4 Hypertensive heart disease	*230 [185-281]*	*185 [151-231]*	*1.2 [0.8-1.9]*	*Ic [Ic-Ic]*	*213 [171-269]*	*208 [172-256]*	*1.0 [0.7-1.6]*	*Ic [Ic-Ic]*
2.2.5 Cardiomyopathy and myocarditis	*162 [123-206]*	*165 [149-201]*	*1.0 [0.6-1.4]*	*Ic [Ib-Ic]*	*174 [122-232]*	*175 [157-186]*	*1.0 [0.7-1.5]*	*Ic [Ib-Ic]*
2.2.6 Atrial fibrillation and flutter	*38 [26-54]*	*135 [104-172]*	*.3 [0.2-0.5]*	*Ia [Ia-Ib]*	*27 [18-39]*	*76 [58-97]*	*.4 [0.2-0.7]*	*Ib [Ia-Ic]*
2.2.7 Aortic aneurysm	*38 [23-57]*	*91 [69-120]*	*.4 [0.2-0.8]*	*Ib [Ia-Ic]*	*34 [17-54]*	*98 [77-123]*	*.3 [0.1-0.7]*	*Ib [Ia-Ic]*
2.2.8 Peripheral vascular disease	*11 [7-17]*	*37 [25-55]*	*.3 [0.1-0.7]*	*Ia [Ia-Ic]*	*7 [4-11]*	*23 [15-34]*	*.3 [0.1-0.7]*	*Ia [Ia-Ic]*
2.2.9 Endocarditis	*24 [17-31]*	*17 [14-20]*	*1.4 [0.9-2.3]*	*Ic [Ic-Ic]*	*30 [22-41]*	*19 [16-22]*	*1.6 [1.0-2.5]*	*Ic [Ic-Ic]*
2.2.10 Other cardiovascular and circulatory diseases	*232 [198-275]*	*338 [298-386]*	*.7 [0.5-0.9]*	*Ic [Ib-Ic]*	*227 [181-283]*	*378 [346-419]*	*.6 [0.4-0.8]*	*Ib [Ib-Ic]*
**2.3 Chronic respiratory diseases**	**1777 [1386-2299]**	**1387 [1068-1795]**	**1.3 [0.8-2.2]**	**Ic [Ic-Ic]**	**2431 [1933-3114]**	**1453 [1148-1853]**	**1.7 [1.0-2.7]**	**Ic [Ic-Ic]**
2.3.1 Chronic obstructive pulmonary disease	*1165 [956-1421]*	*849 [700-1029]*	*1.4 [0.9-2.0]*	*Ic [Ic-Ic]*	*1623 [1404-1887]*	*828 [694-994]*	*2.0 [1.4-2.7]*	*Ic [Ic-Ic]*
2.3.2 Pneumoconiosis	*37 [18-71]*	*42 [28-65]*	*.9 [0.3-2.5]*	*Ic [Ia-Ic]*	*70 [29-136]*	*52 [37-75]*	*1.3 [0.4-3.7]*	*Ic [Ib-II]*
2.3.3 Asthma	*327 [235-467]*	*327 [214-474]*	*1.0 [0.5-2.2]*	*Ic [Ib-Ic]*	*412 [284-607]*	*387 [272-543]*	*1.1 [0.5-2.2]*	*Ic [Ib-Ic]*
2.3.4 Interstitial lung disease and pulmonary sarcoidosis	*25 [17-37]*	*73 [48-102]*	*.3 [0.2-0.8]*	*Ib [Ia-Ic]*	*26 [15-42]*	*47 [35-70]*	*.5 [0.2-1.2]*	*Ib [Ia-Ic]*
2.3.5 Other chronic respiratory diseases	*222 [160-304]*	*97 [77-124]*	*2.3 [1.3-3.9]*	*Ic [Ic-II]*	*300 [202-443]*	*138 [111-171]*	*2.2 [1.2-4.0]*	*Ic [Ic-II]*

Cause or cause group	2010				1990			
	DALYs per 100,000 inhabitants		IRF	Disease Type	DALYs per 100,000 inhabitants		IRF	Disease Type
	LMIC	HIC			LMIC	HIC		
**2.4 Cirrhosis of the liver**	**462 [347-601]**	**394 [320-465]**	**1.2 [0.7-1.9]**	**Ic [Ic-Ic]**	**462 [359-577]**	**464 [388-546]**	**1.0 [0.7-1.5]**	**Ic [Ib-Ic]**
2.4.1 Cirrhosis of the liver secondary to hepatitis B	*143 [109-187]*	*61 [52-76]*	*2.3 [1.4-3.6]*	*Ic [Ic-II]*	*146 [114-179]*	*80 [66-91]*	*1.8 [1.2-2.7]*	*Ic [Ic-Ic]*
2.4.2 Cirrhosis of the liver secondary to hepatitis C	*104 [81-130]*	*133 [110-154]*	*.8 [0.5-1.2]*	*Ic [Ib-Ic]*	*99 [79-120]*	*148 [127-172]*	*.7 [0.5-0.9]*	*Ic [Ib-Ic]*
2.4.3 Cirrhosis of the liver secondary to alcohol use	*120 [86-158]*	*154 [121-183]*	*.8 [0.5-1.3]*	*Ic [Ib-Ic]*	*108 [83-138]*	*181 [149-220]*	*.6 [0.4-0.9]*	*Ib [Ib-Ic]*
2.4.4 Other cirrhosis of the liver	*95 [71-126]*	*45 [37-53]*	*2.1 [1.3-3.4]*	*Ic [Ic-II]*	*109 [83-141]*	*55 [46-63]*	*2.0 [1.3-3.1]*	*Ic [Ic-II]*
**2.5 Digestive diseases except cirrhosis**	**491 [354-700]**	**391 [287-553]**	**1.3 [0.6-2.4]**	**Ic [Ib-Ic]**	**680 [478-922]**	**435 [325-601]**	**1.6 [0.8-2.8]**	**Ic [Ic-Ic]**
2.5.1 Peptic ulcer disease	*108 [86-135]*	*37 [32-47]*	*2.9 [1.8-4.3]*	*Ic [Ic-II]*	*212 [172-241]*	*77 [62-92]*	*2.7 [1.9-3.9]*	*Ic [Ic-II]*
2.5.2 Gastritis and duodenitis	*18 [11-33]*	*13 [9-19]*	*1.3 [0.6-3.6]*	*Ic [Ib-II]*	*25 [15-43]*	*20 [14-29]*	*1.2 [0.5-3.0]*	*Ic [Ib-Ic]*
2.5.3 Appendicitis	*24 [14-38]*	*8 [5-11]*	*3.1 [1.3-7.2]*	*II [Ic-II]*	*41 [24-70]*	*12 [8-17]*	*3.3 [1.4-8.6]*	*II [Ic-II]*
2.5.4 Paralytic ileus and intestinal obstruction without hernia	*58 [39-87]*	*35 [24-45]*	*1.7 [0.9-3.7]*	*Ic [Ic-II]*	*82 [45-119]*	*33 [25-45]*	*2.4 [1.0-4.7]*	*Ic [Ic-II]*
2.5.5 Inguinal or femoral hernia	*12 [7-21]*	*10 [6-19]*	*1.2 [0.4-3.7]*	*Ic [Ib-II]*	*20 [16-30]*	*12 [8-22]*	*1.7 [0.7-3.9]*	*Ic [Ic-II]*
2.5.6 Non-infective inflammatory bowel disease	*37 [24-55]*	*70 [46-110]*	*.5 [0.2-1.2]*	*Ib [Ia-Ic]*	*49 [27-78]*	*75 [51-115]*	*.7 [0.2-1.5]*	*Ib [Ia-Ic]*
2.5.7 Vascular disorders of intestine	*12 [5-28]*	*39 [22-80]*	*.3 [0.1-1.2]*	*Ia [Ia-Ic]*	*12 [5-27]*	*40 [22-81]*	*.3 [0.1-1.2]*	*Ia [Ia-Ic]*
2.5.8 Gall bladder and bile duct disease	*32 [25-41]*	*36 [30-44]*	*.9 [0.6-1.4]*	*Ic [Ib-Ic]*	*41 [31-57]*	*42 [35-49]*	*1.0 [0.6-1.6]*	*Ic [Ib-Ic]*
2.5.9 Pancreatitis	*34 [25-45]*	*36 [28-47]*	*.9 [0.5-1.6]*	*Ic [Ib-Ic]*	*31 [21-42]*	*39 [29-49]*	*.8 [0.4-1.4]*	*Ic [Ib-Ic]*
2.5.10 Other digestive diseases	*156 [117-217]*	*105 [85-131]*	*1.5 [0.9-2.5]*	*Ic [Ic-Ic]*	*166 [121-214]*	*82 [68-102]*	*2.0 [1.2-3.1]*	*Ic [Ic-II]*
**2.6 Neurological disorders**	**1029 [710-1443]**	**1342 [982-1733]**	**.8 [0.4-1.5]**	**Ic [Ib-Ic]**	**909 [612-1294]**	**1007 [743-1324]**	**.9 [0.5-1.7]**	**Ic [Ib-Ic]**
2.6.1 Alzheimer's disease and other dementias	*93 [71-120]*	*583 [441-721]*	*.2 [0.1-0.3]*	*Ia [Ia-Ia]*	*69 [52-89]*	*300 [235-380]*	*.2 [0.1-0.4]*	*Ia [Ia-Ib]*
2.6.2 Parkinson's disease	*18 [13-25]*	*86 [64-110]*	*.2 [0.1-0.4]*	*Ia [Ia-Ib]*	*14 [9-19]*	*57 [45-73]*	*.2 [0.1-0.4]*	*Ia [Ia-Ib]*
2.6.3 Epilepsy	*278 [207-385]*	*116 [92-145]*	*2.4 [1.4-4.2]*	*Ic [Ic-II]*	*282 [206-396]*	*122 [97-152]*	*2.3 [1.4-4.1]*	*Ic [Ic-II]*
2.6.4 Multiple sclerosis	*11 [8-15]*	*41 [33-50]*	*.3 [0.2-0.4]*	*Ia [Ia-Ib]*	*12 [9-17]*	*37 [31-45]*	*.3 [0.2-0.5]*	*Ib [Ia-Ib]*
2.6.5 Migraine	*325 [206-465]*	*332 [215-460]*	*1.0 [0.4-2.2]*	*Ic [Ib-Ic]*	*295 [188-423]*	*343 [222-470]*	*.9 [0.4-1.9]*	*Ic [Ib-Ic]*
2.6.6 Tension-type headache	*25 [15-40]*	*31 [18-48]*	*.8 [0.3-2.2]*	*Ic [Ia-Ic]*	*23 [13-36]*	*30 [18-47]*	*.8 [0.3-2.0]*	*Ic [Ia-Ic]*
2.6.7 Other neurological disorders	*279 [191-394]*	*153 [119-198]*	*1.8 [1.0-3.3]*	*Ic [Ic-II]*	*214 [134-314]*	*119 [94-157]*	*1.8 [0.9-3.4]*	*Ic [Ic-II]*
**2.7 Mental and behavioural disorders**	**2619 [1726-3753]**	**3156 [2155-4394]**	**.8 [0.4-1.7]**	**Ic [Ib-Ic]**	**2470 [1618-3564]**	**3004 [2042-4199]**	**.8 [0.4-1.7]**	**Ic [Ib-Ic]**
2.7.1 Schizophrenia	*215 [131-312]*	*238 [148-338]*	*.9 [0.4-2.1]*	*Ic [Ib-Ic]*	*193 [119-279]*	*227 [142-319]*	*.9 [0.4-2.0]*	*Ic [Ib-Ic]*
2.7.2 Alcohol use disorders	*244 [163-349]*	*333 [237-461]*	*.7 [0.4-1.5]*	*Ic [Ib-Ic]*	*234 [155-333]*	*327 [233-448]*	*.7 [0.3-1.4]*	*Ic [Ib-Ic]*
2.7.3 Drug use disorders	*260 [157-409]*	*472 [315-667]*	*.6 [0.2-1.3]*	*Ib [Ia-Ic]*	*220 [129-358]*	*400 [261-585]*	*.5 [0.2-1.4]*	*Ib [Ia-Ic]*
2.7.3.1 Opioid use disorders	119 [76-177]	219 [154-291]	.5 [0.3-1.2]	Ib [Ia-Ic]	90 [56-138]	152 [106-207]	.6 [0.3-1.3]	Ib [Ia-Ic]
2.7.3.2 Cocaine use disorders	11 [6-19]	43 [24-72]	.3 [0.1-0.8]	Ia [Ia-Ic]	11 [6-18]	43 [24-71]	.3 [0.1-0.7]	Ia [Ia-Ic]
2.7.3.3 Amphetamine use disorders	39 [20-65]	35 [19-56]	1.1 [0.4-3.5]	Ic [Ib-II]	36 [19-61]	39 [21-64]	.9 [0.3-2.9]	Ic [Ia-Ic]
2.7.3.4 Cannabis use disorders	25 [15-41]	56 [35-82]	.5 [0.2-1.2]	Ib [Ia-Ic]	26 [15-42]	63 [40-94]	.4 [0.2-1.0]	Ib [Ia-Ic]
2.7.3.5 Other drug use disorders	66 [40-106]	119 [83-167]	.6 [0.2-1.3]	Ib [Ia-Ic]	57 [33-99]	102 [69-149]	.6 [0.2-1.4]	Ib [Ia-Ic]

Cause or cause group	2010				1990			
	DALYs per 100,000 inhabitants		IRF	Disease Type	DALYs per 100,000 inhabitants		IRF	Disease Type
	LMIC	HIC			LMIC	HIC		
2.7.4 Unipolar depressive disorders	*1074 [753-1450]*	*1129 [806-1503]*	*1.0 [0.5-1.8]*	*Ic [Ib-Ic]*	*1021 [714-1387]*	*1057 [754-1401]*	*1.0 [0.5-1.8]*	*Ic [Ib-Ic]*
2.7.4.1 Major depressive disorder	915 [649-1227]	956 [693-1260]	1.0 [0.5-1.8]	Ic [Ib-Ic]	875 [619-1184]	889 [643-1166]	1.0 [0.5-1.8]	Ic [Ib-Ic]
2.7.4.2 Dysthymia	159 [103-223]	173 [113-242]	.9 [0.4-2.0]	Ic [Ib-Ic]	146 [95-204]	169 [111-235]	.9 [0.4-1.8]	Ic [Ib-Ic]
2.7.5 Bipolar affective disorder	*188 [117-279]*	*182 [114-268]*	*1.0 [0.4-2.5]*	*Ic [Ib-Ic]*	*171 [106-253]*	*188 [118-278]*	*.9 [0.4-2.1]*	*Ic [Ib-Ic]*
2.7.6 Anxiety disorders	*372 [243-550]*	*502 [345-706]*	*.7 [0.3-1.6]*	*Ic [Ib-Ic]*	*346 [224-516]*	*513 [352-728]*	*.7 [0.3-1.5]*	*Ic [Ia-Ic]*
2.7.7 Eating disorders	*21 [13-35]*	*90 [57-138]*	*.2 [0.1-0.6]*	*Ia [Ia-Ib]*	*16 [9-26]*	*66 [42-103]*	*.2 [0.1-0.6]*	*Ia [Ia-Ib]*
2.7.8 Pervasive develpment disorders	*112 [74-161]*	*110 [74-156]*	*1.0 [0.5-2.2]*	*Ic [Ib-Ic]*	*113 [75-162]*	*112 [75-159]*	*1.0 [0.5-2.2]*	*Ic [Ib-Ic]*
2.7.8.1 Autism	58 [39-83]	58 [40-81]	1.0 [0.5-2.1]	Ic [Ib-Ic]	59 [39-84]	58 [40-82]	1.0 [0.5-2.1]	Ic [Ib-Ic]
2.7.8.2 Asperger's syndrome	54 [35-77]	52 [35-75]	1.0 [0.5-2.2]	Ic [Ib-Ic]	54 [35-78]	53 [35-77]	1.0 [0.5-2.2]	Ic [Ib-Ic]
2.7.9 Childhood behavioural disorders	*96 [55-152]*	*60 [34-93]*	*1.6 [0.6-4.4]*	*Ic [Ib-II]*	*111 [63-176]*	*71 [41-112]*	*1.6 [0.6-4.3]*	*Ic [Ib-II]*
2.7.9.1 Attention-deficit hyperactivity disorder	8 [4-12]	5 [3-8]	1.6 [0.5-4.5]	Ic [Ib-II]	9 [5-14]	6 [3-9]	1.5 [0.5-4.2]	Ic [Ib-II]
2.7.9.2 Conduct disorder	89 [51-139]	55 [31-85]	1.6 [0.6-4.4]	Ic [Ib-II]	102 [58-163]	65 [37-103]	1.6 [0.6-4.4]	Ic [Ib-II]
2.7.10 Idiopathic intellectual disability	*16 [7-28]*	*11 [6-20]*	*1.4 [0.4-5.0]*	*Ic [Ib-II]*	*25 [13-41]*	*18 [10-28]*	*1.4 [0.5-4.2]*	*Ic [Ib-II]*
2.7.11 Other mental and behavioral disorders	*20 [13-30]*	*30 [19-44]*	*.7 [0.3-1.6]*	*Ic [Ia-Ic]*	*21 [12-31]*	*26 [16-38]*	*.8 [0.3-1.9]*	*Ic [Ia-Ic]*
**2.8 Diabetes,urogenital, blood and endocrine diseases**	**1761 [1255-2495]**	**1916 [1380-2780]**	**.9 [0.5-1.8]**	**Ic [Ib-Ic]**	**1626 [1098-2536]**	**1577 [1152-2211]**	**1.0 [0.5-2.2]**	**Ic [Ib-Ic]**
2.8.1 Diabetes mellitus	*676 [555-819]*	*720 [588-900]*	*.9 [0.6-1.4]*	*Ic [Ib-Ic]*	*506 [419-623]*	*629 [518-778]*	*.8 [0.5-1.2]*	*Ic [Ib-Ic]*
2.8.2 Acute glomerulonephritis	*63 [22-155]*	*1 [0-1]*	*100.5 [22.2-491.6]*	*III [II-III]*	*155 [46-462]*	*1 [1-2]*	*130.5 [22.5-622.8]*	*III [II-III]*
2.8.3 Chronic kidney diseases	*302 [248-352]*	*344 [288-407]*	*.9 [0.6-1.2]*	*Ic [Ib-Ic]*	*263 [212-324]*	*277 [235-329]*	*.9 [0.6-1.4]*	*Ic [Ib-Ic]*
2.8.3.1 Chronic kidney disease due to diabetes mellitus	60 [49-72]	112 [93-133]	.5 [0.4-0.8]	Ib [Ib-Ic]	43 [35-54]	86 [72-103]	.5 [0.3-0.7]	Ib [Ib-Ic]
2.8.3.2 Chronic kidney disease due to hypertension	66 [55-76]	74 [63-86]	.9 [0.6-1.2]	Ic [Ib-Ic]	53 [44-64]	58 [50-68]	.9 [0.6-1.3]	Ic [Ib-Ic]
2.8.3.3 Chronic kidney disease unspecified	176 [145-204]	158 [132-187]	1.1 [0.8-1.5]	Ic [Ic-Ic]	166 [132-205]	134 [113-159]	1.2 [0.8-1.8]	Ic [Ic-Ic]
2.8.4 Urinary diseases and male infertility	*166 [100-275]*	*373 [213-591]*	*.4 [0.2-1.3]*	*Ib [Ia-Ic]*	*136 [77-227]*	*247 [154-386]*	*.5 [0.2-1.5]*	*Ib [Ia-Ic]*
2.8.4.1 Tubulointerstitial nephritis, pyelonephritis, and urinary tract infections	41 [28-56]	68 [40-90]	.6 [0.3-1.4]	Ib [Ia-Ic]	39 [24-57]	42 [34-59]	.9 [0.4-1.7]	Ic [Ib-Ic]
2.8.4.2 Urolithiasis	16 [10-35]	15 [10-23]	1.1 [0.4-3.7]	Ic [Ib-II]	18 [11-32]	14 [9-20]	1.3 [0.5-3.4]	Ic [Ib-II]
2.8.4.3 Benign prostatic hyperplasia	72 [39-125]	260 [141-436]	.3 [0.1-0.9]	Ia [Ia-Ic]	51 [27-90]	170 [96-280]	.3 [0.1-0.9]	Ia [Ia-Ic]
2.8.4.4 Male infertility	3 [1-6]	1 [0-2]	2.4 [0.4-13.6]	Ic [Ib-II]	3 [1-6]	1 [0-3]	2.1 [0.4-12.5]	Ic [Ib-II]
2.8.4.5 Other urinary diseases	34 [22-52]	29 [22-40]	1.2 [0.5-2.4]	Ic [Ib-Ic]	26 [14-42]	20 [15-24]	1.3 [0.6-2.9]	Ic [Ib-Ic]
2.8.5 Gynaecological disorders	*155 [72-290]*	*121 [47-243]*	*1.3 [0.3-6.2]*	*Ic [Ia-II]*	*154 [75-283]*	*126 [49-256]*	*1.2 [0.3-5.8]*	*Ic [Ia-II]*
2.8.5.1 Uterine fibroids	47 [30-72]	31 [14-58]	1.5 [0.5-5.0]	Ic [Ib-II]	48 [32-71]	27 [13-51]	1.8 [0.6-5.5]	Ic [Ib-II]

Cause or cause group	2010				1990			
	DALYs per 100,000 inhabitants		IRF	Disease Type	DALYs per 100,000 inhabitants		IRF	Disease Type
	LMIC	HIC			LMIC	HIC		
2.8.5.2 Polycystic ovarian syndrome	41 [19-79]	34 [16-63]	1.2 [0.3-5.0]	Ic [Ia-II]	38 [18-73]	39 [19-74]	1.0 [0.2-3.9]	Ic [Ia-II]
2.8.5.3 Female infertility	2 [1-4]	1 [0-2]	2.4 [0.4-13.6]	Ic [Ib-II]	2 [1-4]	1 [0-2]	2.0 [0.4-11.5]	Ic [Ib-II]
2.8.5.4 Endometriosis	8 [3-16]	7 [2-13]	1.2 [0.2-6.6]	Ic [Ia-II]	8 [3-15]	8 [3-15]	1.0 [0.2-5.4]	Ic [Ia-II]
2.8.5.5 Genital prolapse	26 [10-53]	29 [12-59]	.9 [0.2-4.5]	Ic [Ia-II]	25 [10-52]	29 [12-60]	.8 [0.2-4.3]	Ic [Ia-II]
2.8.5.6 Premenstrual syndrome	18 [1-49]	17 [1-46]	1.1 [0.0-55.5]	Ic [Ia-III]	18 [1-48]	20 [1-53]	.9 [0.0-47.1]	Ic [Ia-III]
2.8.5.7 Other gynecological diseases	12 [8-16]	2 [2-3]	5.8 [3.2-10.3]	II [II-II]	15 [10-21]	2 [1-2]	8.5 [4.5-15.7]	II [II-II]
2.8.6 Haemoglobinopathies and haemolytic anaemias	*240 [161-352]*	*161 [103-258]*	*1.5 [0.6-3.4]*	*Ic [Ib-II]*	*291 [193-440]*	*179 [113-287]*	*1.6 [0.7-3.9]*	*Ic [Ic-II]*
2.8.6.1 Thalassemias	89 [64-123]	51 [35-72]	1.7 [0.9-3.5]	Ic [Ic-II]	111 [80-152]	59 [42-83]	1.9 [1.0-3.6]	Ic [Ic-II]
2.8.6.2 Sickle cell disorders	81 [53-115]	91 [54-159]	.9 [0.3-2.1]	Ic [Ib-Ic]	80 [54-114]	96 [55-172]	.8 [0.3-2.1]	Ic [Ia-Ic]
2.8.6.3 G6PD deficiency	4 [3-6]	6 [4-9]	.8 [0.3-1.7]	Ic [Ia-Ic]	6 [4-9]	8 [5-13]	.8 [0.3-1.9]	Ic [Ia-Ic]
2.8.6.4 Other hemoglobinopathies and hemolytic anemias	66 [40-109]	13 [10-18]	5.1 [2.3-10.6]	II [Ic-II]	94 [56-165]	16 [11-20]	6.0 [2.9-14.5]	II [Ic-II]
2.8.7 Other endocrine, nutritional, blood, and immune disorders	*160 [98-253]*	*197 [140-379]*	*.8 [0.3-1.8]*	*Ic [Ia-Ic]*	*122 [76-177]*	*118 [82-172]*	*1.0 [0.4-2.2]*	*Ic [Ib-Ic]*
**2.9 Musculoskeletal disorders**	**2247 [1577-3068]**	**3762 [2639-5084]**	**.6 [0.3-1.2]**	**Ib [Ia-Ic]**	**1967 [1364-2690]**	**3447 [2414-4664]**	**.6 [0.3-1.1]**	**Ib [Ia-Ic]**
2.9.1 Rheumatoid arthritis	*55 [40-75]*	*156 [112-207]*	*.4 [0.2-0.7]*	*Ib [Ia-Ic]*	*48 [34-65]*	*140 [101-182]*	*.3 [0.2-0.6]*	*Ib [Ia-Ib]*
2.9.2 Osteoarthritis	*235 [156-342]*	*333 [206-506]*	*.7 [0.3-1.7]*	*Ic [Ia-Ic]*	*184 [121-269]*	*270 [166-411]*	*.7 [0.3-1.6]*	*Ic [Ia-Ic]*
2.9.3 Low back and neck pain	*1568 [1064-2195]*	*2465 [1669-3415]*	*.6 [0.3-1.3]*	*Ib [Ia-Ic]*	*1407 [943-1970]*	*2326 [1576-3223]*	*.6 [0.3-1.2]*	*Ib [Ia-Ic]*
2.9.3.1 Low back pain	1118 [762-1546]	1743 [1178-2407]	.6 [0.3-1.3]	Ib [Ia-Ic]	1002 [673-1390]	1631 [1106-2239]	.6 [0.3-1.3]	Ib [Ia-Ic]
2.9.3.2 Neck pain	450 [302-649]	722 [490-1009]	.6 [0.3-1.3]	Ib [Ia-Ic]	405 [270-579]	695 [470-984]	.6 [0.3-1.2]	Ib [Ia-Ic]
2.9.4 Gout	*1 [0-1]*	*7 [4-10]*	*.1 [0.0-0.3]*	*Ia [Ia-Ia]*	*1 [0-1]*	*5 [3-8]*	*.1 [0.0-0.3]*	*Ia [Ia-Ia]*
2.9.5 Other musculoskeletal disorders	*389 [317-454]*	*801 [649-945]*	*.5 [0.3-0.7]*	*Ib [Ib-Ic]*	*328 [265-385]*	*706 [567-839]*	*.5 [0.3-0.7]*	*Ib [Ia-Ic]*
**2.10 Other non-communicable diseases**	**1930 [1092-3180]**	**1441 [787-2443]**	**1.3 [0.4-4.0]**	**Ic [Ib-II]**	**2504 [1401-4232]**	**1705 [974-2759]**	**1.5 [0.5-4.3]**	**Ic [Ib-II]**
2.10.1 Congenital anomalies	*628 [399-937]*	*214 [164-281]*	*2.9 [1.4-5.7]*	*Ic [Ic-II]*	*1160 [687-1912]*	*399 [302-490]*	*2.9 [1.4-6.3]*	*Ic [Ic-II]*
2.10.1.1 Neural tube defects	107 [52-169]	10 [8-15]	10.4 [3.5-22.0]	II [II-II]	227 [110-368]	40 [28-51]	5.7 [2.1-13.3]	II [Ic-II]
2.10.1.2 Congenital heart anomalies	250 [198-330]	83 [67-106]	3.0 [1.9-4.9]	II [Ic-II]	459 [363-652]	192 [154-219]	2.4 [1.7-4.2]	Ic [Ic-II]
2.10.1.3 Cleft lip and cleft palate	9 [6-15]	2 [1-3]	5.6 [2.2-15.1]	II [Ic-II]	22 [11-44]	2 [2-3]	9.7 [3.3-29.2]	II [II-II]
2.10.1.4 Down's syndrome	26 [16-41]	22 [16-29]	1.2 [0.5-2.5]	Ic [Ib-Ic]	44 [20-78]	21 [16-29]	2.1 [0.7-5.0]	Ic [Ic-II]
2.10.1.5 Other chromosomal abnormalities	26 [13-52]	21 [15-28]	1.3 [0.5-3.4]	Ic [Ib-II]	64 [20-166]	27 [19-37]	2.4 [0.5-8.5]	Ic [Ib-II]
2.10.1.6 Other congenital anomalies	208 [115-329]	76 [57-100]	2.7 [1.1-5.8]	Ic [Ic-II]	343 [163-603]	116 [84-149]	2.9 [1.1-7.2]	Ic [Ic-II]
2.10.2 Skin and subcutaneous diseases	*538 [258-959]*	*539 [248-979]*	*1.0 [0.3-3.9]*	*Ic [Ia-II]*	*581 [286-1019]*	*539 [247-988]*	*1.1 [0.3-4.1]*	*Ic [Ia-II]*
2.10.2.1 Eczema	129 [63-209]	134 [65-221]	1.0 [0.3-3.2]	Ic [Ia-II]	130 [65-212]	135 [64-227]	1.0 [0.3-3.3]	Ic [Ia-II]
2.10.2.2 Psoriasis	14 [7-23]	23 [11-36]	.6 [0.2-2.1]	Ib [Ia-Ic]	13 [6-20]	21 [11-34]	.6 [0.2-1.9]	Ib [Ia-Ic]

Cause or cause group	2010				1990			
	DALYs per 100,000 inhabitants		IRF	Disease Type	DALYs per 100,000 inhabitants		IRF	Disease Type
	LMIC	HIC			LMIC	HIC		
2.10.2.3 Cellulitis	20 [13-33]	10 [5-24]	1.9 [0.5-6.3]	Ic [Ib-II]	31 [19-47]	11 [6-26]	2.9 [0.8-8.4]	Ic [Ic-II]
2.10.2.4 Abscess, impetigo, and other bacterial skin diseases	46 [31-70]	16 [10-26]	2.9 [1.2-7.2]	Ic [Ic-II]	69 [45-101]	16 [10-27]	4.3 [1.7-9.9]	II [Ic-II]
2.10.2.5 Scabies	26 [12-47]	7 [3-13]	3.5 [0.9-13.6]	II [Ic-II]	41 [20-76]	9 [4-16]	4.7 [1.2-18.0]	II [Ic-II]
2.10.2.6 Fungal skin diseases	35 [11-82]	27 [8-61]	1.3 [0.2-9.7]	Ic [Ia-II]	32 [10-74]	25 [8-59]	1.2 [0.2-9.1]	Ic [Ia-II]
2.10.2.7 Viral skin diseases	40 [17-74]	40 [17-74]	1.0 [0.2-4.4]	Ic [Ia-II]	45 [20-83]	43 [18-82]	1.0 [0.2-4.6]	Ic [Ia-II]
2.10.2.8 Acne vulgaris	57 [26-110]	63 [29-123]	.9 [0.2-3.8]	Ic [Ia-II]	60 [27-115]	75 [34-148]	.8 [0.2-3.3]	Ic [Ia-II]
2.10.2.9 Alopecia areata	20 [6-38]	20 [6-39]	1.0 [0.2-6.0]	Ic [Ia-II]	19 [6-36]	20 [6-39]	.9 [0.2-5.7]	Ic [Ia-II]
2.10.2.10 Pruritus	27 [13-52]	49 [23-93]	.5 [0.1-2.3]	Ib [Ia-Ic]	24 [11-45]	45 [21-84]	.5 [0.1-2.2]	Ib [Ia-Ic]
2.10.2.11 Urticaria	38 [14-67]	37 [14-65]	1.0 [0.2-4.7]	Ic [Ia-II]	37 [14-66]	37 [15-66]	1.0 [0.2-4.5]	Ic [Ia-II]
2.10.2.12 Decubitus ulcer	17 [12-24]	22 [13-35]	.8 [0.3-1.8]	Ic [Ia-Ic]	18 [13-26]	19 [12-31]	1.0 [0.4-2.1]	Ic [Ib-Ic]
2.10.2.13 Other skin and subcutaneous diseases	69 [32-129]	90 [42-167]	.8 [0.2-3.1]	Ic [Ia-II]	63 [30-117]	81 [38-149]	.8 [0.2-3.1]	Ic [Ia-II]
2.10.3 Sense organ diseases	*519 [330-802]*	*431 [249-716]*	*1.2 [0.5-3.2]*	*Ic [Ib-II]*	*489 [311-748]*	*426 [249-705]*	*1.1 [0.4-3.0]*	*Ic [Ib-II]*
2.10.3.1 Glaucoma	14 [10-19]	11 [8-15]	1.3 [0.7-2.4]	Ic [Ic-Ic]	9 [6-12]	8 [5-11]	1.1 [0.6-2.2]	Ic [Ib-Ic]
2.10.3.2 Cataracts	76 [56-100]	30 [21-42]	2.5 [1.3-4.7]	Ic [Ic-II]	88 [66-116]	42 [30-59]	2.1 [1.1-3.9]	Ic [Ic-II]
2.10.3.3 Macular degeneration	19 [14-26]	20 [14-27]	1.0 [0.5-1.9]	Ic [Ib-Ic]	9 [7-13]	12 [8-16]	.8 [0.4-1.6]	Ic [Ib-Ic]
2.10.3.4 Refraction and accommodation disorders	92 [66-126]	20 [15-27]	4.5 [2.5-8.4]	II [Ic-II]	79 [57-107]	18 [13-24]	4.4 [2.4-8.1]	II [Ic-II]
2.10.3.5 Other hearing loss	233 [139-376]	210 [123-342]	1.1 [0.4-3.0]	Ic [Ib-II]	233 [138-372]	227 [133-367]	1.0 [0.4-2.8]	Ic [Ib-Ic]
2.10.3.6 Other vision loss	83 [43-150]	136 [66-257]	.6 [0.2-2.3]	Ib [Ia-Ic]	69 [36-124]	118 [58-223]	.6 [0.2-2.1]	Ib [Ia-Ic]
2.10.3.7 Other sense organ diseases	2 [1-4]	2 [1-5]	.8 [0.1-5.2]	Ic [Ia-II]	2 [1-4]	2 [1-5]	.8 [0.1-5.5]	Ic [Ia-II]
2.10.4 Oral disorders	*217 [93-419]*	*227 [109-414]*	*1.0 [0.2-3.9]*	*Ic [Ia-II]*	*230 [103-434]*	*268 [135-473]*	*.9 [0.2-3.2]*	*Ic [Ia-II]*
2.10.4.1 Dental caries	*81 [33-156]*	*24 [10-47]*	*3.3 [0.7-15.4]*	*II [Ic-II]*	*80 [32-156]*	*25 [10-47]*	*3.2 [0.7-15.1]*	*II [Ic-II]*
2.10.4.2 Periodontal disease	77 [28-167]	88 [32-188]	.9 [0.1-5.2]	Ic [Ia-II]	63 [23-136]	78 [29-166]	.8 [0.1-4.7]	Ic [Ia-II]
2.10.4.3 Edentulism	59 [32-96]	115 [66-179]	.5 [0.2-1.5]	Ib [Ia-Ic]	87 [47-142]	165 [95-259]	.5 [0.2-1.5]	Ib [Ia-Ic]
2.10.5 Sudden infant death syndrome	27 [11-62]	30 [17-52]	.9 [0.2-3.7]	Ic [Ia-II]	44 [15-119]	73 [41-105]	.6 [0.1-2.9]	Ib [Ia-Ic]
**3. Injuries**	**4358 [3087-6117]**	**2535 [1937-3246]**	**1.7 [1.0-3.2]**	**Ic [Ic-II]**	**5020 [3514-6954]**	**3104 [2466-3905]**	**1.6 [0.9-2.8]**	**Ic [Ic-Ic]**
**3.1 Transport injuries**	**1295 [946-1773]**	**701 [570-874]**	**1.8 [1.1-3.1]**	**Ic [Ic-II]**	**1200 [797-1705]**	**1060 [873-1278]**	**1.1 [0.6-2.0]**	**Ic [Ib-Ic]**
3.1.1 Road injury	*1203 [880-1649]*	*631 [514-783]*	*1.9 [1.1-3.2]*	*Ic [Ic-II]*	*1114 [737-1586]*	*992 [817-1189]*	*1.1 [0.6-1.9]*	*Ic [Ib-Ic]*
3.1.1.1 Pedestrian injury by road vehicle	421 [307-588]	103 [82-127]	4.1 [2.4-7.2]	II [Ic-II]	367 [245-506]	161 [131-207]	2.3 [1.2-3.9]	Ic [Ic-II]
3.1.1.2 Pedal cycle vehicle	73 [51-98]	40 [32-50]	1.8 [1.0-3.1]	Ic [Ic-II]	66 [46-90]	53 [41-63]	1.3 [0.7-2.2]	Ic [Ic-Ic]

Cause or cause group	2010				1990			
	DALYs per 100,000 inhabitants		IRF	Disease Type	DALYs per 100,000 inhabitants		IRF	Disease Type
	LMIC	HIC			LMIC	HIC		
3.1.1.3 Motorized vehicle with two wheels	193 [145-245]	97 [76-119]	2.0 [1.2-3.2]	Ic [Ic-II]	160 [111-219]	185 [155-212]	.9 [0.5-1.4]	Ic [Ib-Ic]
3.1.1.4 Motorized vehicle with three or more wheels	416 [328-517]	385 [320-480]	1.1 [0.7-1.6]	Ic [Ic-Ic]	372 [266-487]	585 [484-695]	.6 [0.4-1.0]	Ib [Ib-Ic]
3.1.1.5 Road injury other	101 [48-202]	5 [4-7]	18.6 [6.5-52.8]	II [II-III]	148 [69-284]	9 [6-12]	16.2 [5.5-44.9]	II [II-III]
3.1.2 Other transport injury	*92 [67-123]*	*70 [55-91]*	*1.3 [0.7-2.2]*	*Ic [Ic-Ic]*	*86 [60-119]*	*68 [56-89]*	*1.3 [0.7-2.1]*	*Ic [Ic-Ic]*
**3.2 Unintentional injuries other than transport injuries**	**1872 [1351-2549]**	**1128 [850-1482]**	**1.7 [0.9-3.0]**	**Ic [Ic-Ic]**	**2722 [1949-3725]**	**1182 [922-1517]**	**2.3 [1.3-4.0]**	**Ic [Ic-II]**
3.2.1 Falls	*490 [378-617]*	*665 [505-878]*	*.7 [0.4-1.2]*	*Ic [Ib-Ic]*	*478 [373-612]*	*563 [424-742]*	*.8 [0.5-1.4]*	*Ic [Ib-Ic]*
3.2.2 Drowning	*326 [260-437]*	*68 [54-84]*	*4.8 [3.1-8.0]*	*II [II-II]*	*636 [477-802]*	*105 [86-129]*	*6.0 [3.7-9.3]*	*II [II-II]*
3.2.3 Fire, heat and hot substances	*316 [200-451]*	*55 [44-74]*	*5.7 [2.7-10.3]*	*II [Ic-II]*	*376 [283-483]*	*75 [61-92]*	*5.0 [3.1-7.9]*	*II [II-II]*
3.2.5 Exposure to mechanical forces	*191 [112-288]*	*54 [41-82]*	*3.5 [1.4-7.0]*	*II [Ic-II]*	*348 [190-586]*	*102 [78-138]*	*3.4 [1.4-7.5]*	*II [Ic-II]*
3.2.4 Poisonings	*141 [98-201]*	*68 [29-93]*	*2.1 [1.1-6.8]*	*Ic [Ic-II]*	*242 [161-402]*	*65 [46-95]*	*3.7 [1.7-8.7]*	*II [Ic-II]*
3.2.5.1 Mechanical forces (firearm)	*77 [46-123]*	*11 [8-20]*	*7.0 [2.3-15.6]*	*II [Ic-II]*	*168 [93-283]*	*28 [18-38]*	*6.1 [2.4-15.3]*	*II [Ic-II]*
3.2.5.2 Mechanical forces (other)	114 [66-165]	43 [33-63]	2.7 [1.1-5.0]	Ic [Ic-II]	179 [97-304]	74 [60-100]	2.4 [1.0-5.1]	Ic [Ic-II]
3.2.6 Adverse effects of medical treatment	60 [42-80]	59 [46-77]	1.0 [0.6-1.7]	Ic [Ib-Ic]	48 [33-66]	44 [35-53]	1.1 [0.6-1.9]	Ic [Ib-Ic]
3.2.7 Animal contact	*62 [31-113]*	*5 [3-6]*	*13.6 [5.0-34.8]*	*II [II-II]*	*107 [53-185]*	*9 [7-13]*	*11.2 [4.0-27.7]*	*II [II-II]*
3.2.7.1 Animal contact (venomous)	46 [24-87]	2 [2-3]	19.4 [6.9-52.3]	II [II-III]	80 [42-134]	5 [4-8]	15.3 [5.6-37.8]	II [II-III]
3.2.7.2 Animal contact (non-venomous)	16 [8-27]	2 [2-3]	7.2 [2.7-16.8]	II [Ic-II]	27 [11-52]	4 [3-6]	6.3 [2.0-16.5]	II [Ic-II]
3.2.8 Unintentional injuries not classified elsewhere	*287 [229-362]*	*154 [126-186]*	*1.9 [1.2-2.9]*	*Ic [Ic-Ic]*	*488 [380-588]*	*218 [184-255]*	*2.2 [1.5-3.2]*	*Ic [Ic-II]*
**3.3 Self-harm and interpersonal violence**	**948 [647-1315]**	**690 [508-865]**	**1.4 [0.7-2.6]**	**Ic [Ic-Ic]**	**957 [670-1300]**	**848 [663-1088]**	**1.1 [0.6-2.0]**	**Ic [Ib-Ic]**
3.3.1 Self-harm	*537 [367-747]*	*520 [376-634]*	*1.0 [0.6-2.0]*	*Ic [Ib-Ic]*	*555 [401-736]*	*605 [483-783]*	*.9 [0.5-1.5]*	*Ic [Ib-Ic]*
3.3.2 Interpersonal violence	*411 [280-568]*	*170 [132-231]*	*2.4 [1.2-4.3]*	*Ic [Ic-II]*	*402 [270-564]*	*244 [180-305]*	*1.7 [0.9-3.1]*	*Ic [Ic-II]*
3.3.2.1 Assault by firearm	174 [120-225]	96 [75-123]	1.8 [1.0-3.0]	Ic [Ic-Ic]	161 [112-227]	135 [101-167]	1.2 [0.7-2.3]	Ic [Ic-Ic]
3.3.2.2 Assault by sharp object	114 [73-173]	40 [29-62]	2.9 [1.2-6.1]	Ic [Ic-II]	99 [62-153]	50 [33-70]	2.0 [0.9-4.6]	Ic [Ic-II]
3.3.2.3 Assault by other means	123 [86-170]	34 [28-46]	3.6 [1.9-6.1]	II [Ic-II]	142 [96-185]	58 [46-68]	2.4 [1.4-4.0]	Ic [Ic-II]
**3.4 Forces of nature, war, and legal intervention**	**243 [142-481]**	**17 [10-26]**	**14.7 [5.6-46.2]**	**II [II-III]**	**141 [98-223]**	**14 [8-22]**	**10.2 [4.4-27.1]**	**II [II-II]**
3.4.1 Exposure to forces of nature	*226 [131-455]*	*16 [10-24]*	*14.4 [5.4-46.1]*	*II [II-III]*	*38 [24-68]*	*2 [1-3]*	*25.2 [9.4-80.2]*	*II [II-III]*
3.4.2 Collective violence and legal intervention	*16 [12-26]*	*1 [1-1]*	*18.7 [8.5-48.8]*	*II [II-III]*	*103 [74-155]*	*12 [7-20]*	*8.4 [3.8-21.0]*	*II [II-II]*
