# Supplementary material for: Poverty-related and neglected diseases – an economic and epidemiological analysis of poverty relatedness and neglect in research and development
Source: Glob Health Action. 2015 Jan 22;8:10.3402/gha.v8.25818. doi: 10.3402/gha.v8.25818 (PMC4306754; doi:10.3402/gha.v8.25818)
Supplement: Poverty-related and neglected diseases – an economic and epidemiological analysis of poverty relatedness and neglect in research and development [file GHA-8-25818-s003.pdf]

Supplementary file 1 – Methodological annexe

This file is supplementary to: von Philipsborn, Peter; Steinbeis, Fridolin; Bender, Max E.; Regmi, Sadie; Tinnemann, Peter: *Poverty-related and neglected diseases: an economic and epidemiological analysis of poverty relatedness and neglect in research and development.* In: Global Health Action 2014, 7: 25818; http://dx.doi.org/10.3402/gha.v7.25818

Correspondence to: Peter von Philipsborn, Faculty of Medicine, Technische Universität München, Stuntzstraße 12, DE-81667 München, Germany, Email: peter.philipsborn@alumni.lse.ac.uk

This work was supported by the German Research Foundation (DFG) and the Technische Universität München within the funding programme Open Access Publishing.

Methodological annexe

All calculations were done with IBM SPSS Statistics®. Syntax and data files are available from the authors on request.

Relatedness of diseases to the level of economic development

All disease burden data is based on the Global Burden of Disease Study 2010 (GBD 2010). GBD 2010 data are provided by the Institute for Health Metrics and Evaluation (IHME) at: http://ghdx.healthmetricsandevaluation.org/global-burden-disease-study-2010-gbd-2010-data-download (Accessed 01.02.2014).

In our calculation of the income relation factor (IRF) we followed GBD 2010 in classifying the following regions as high-income regions: Southern Latin America, Western Europe, High-income North America, Australasia, and High-income Asia Pacific. The remaining regions were classified as low and middle income countries (LMIC). (1)

We calculated absolute DALY figures for HIC and LMIC by adding up absolute DALY figures included in the GBD 2010 data set for the respective regions. We calculated figures for "DALYs per 100,000 inhabitants in LMIC" and "DALYs per 100,000 inhabitants in HIC" using population figures provided by the World Bank at: http://data.worldbank.org/indicator/SP.POP.TOTL
(Accessed 01.02.2014).

Population figures for HIC and LMIC were calculated by summing up national population figures of all countries belong to the respective regions as defined by GBD 2010 and as described above.

The following population figures were used:

Income category	1990	2005	2010
high-income countries	886,419,803	984,709,143	1,017,132,480
low-income countries	4,378,229,929	5,489,963,781	5,851,839,259
WORLD	5,278,928,953	6,490,741,058	6,885,217,727

The following formula were used:

$$DALYs\ per\ 100{,}000\ inhabitants\ in\ low\ and\ middle\ income\ countries =$$

$$= \frac{Sum\ of\ absolute\ DALYs\ in\ all\ low\ and\ middle\ income\ regions}{Sum\ of\ population\ of\ all\ countries\ belonging\ to\ these\ regions} \times\ 100{,}000$$

$$DALYs\ per\ 100{,}000\ inhabitants\ in\ high\ income\ countries =$$

$$= \frac{Sum\ of\ absolute\ DALYs\ in\ all\ high\ income\ regions}{Sum\ of\ population\ of\ all\ high\ income\ countries} \times\ 100{,}000$$

The Income Relation Factor (IRF) was then calculated using the following ratio:

$$IRF = \frac{DALYs\ per\ 100{,}000\ inhabitants\ in\ low\ and\ middle\ income\ countries}{DALYs\ per\ 100{,}000\ inhabitants\ in\ high\ income\ countries}$$

Calculation of uncertainty intervals

The calculation of the uncertainty intervals was based on the 95% uncertainty intervals reported by GBD 2010. To calculate the uncertainty intervals for the IRF, lower and upper bound estimates for LMIC and HIC countries were combined to yield figures reflecting the 95% uncertainty of the original DALY data:

$$IRF\ (lower\ bound\ estimate) =$$

$$= \frac{DALYs\ per\ 100{,}000\ inhabitants\ in\ low\ and\ middle\ income\ countries\ (lower\ bound\ estimate)}{DALYs\ per\ 100{,}000\ inhabitants\ in\ high\ income\ countries\ (upper\ bound\ estimate)}$$

$$IRF\ (upper\ bound\ estimate) =$$

$$= \frac{DALYs\ per\ 100{,}000\ inhabitants\ in\ low\ and\ middle\ income\ countries\ (upper\ bound\ estimate)}{DALYs\ per\ 100{,}000\ inhabitants\ in\ high\ income\ countries\ (lower\ bound\ estimate)}$$

R&D Expenditure and Neglect in R&D

For figures on total world-wide health-related R&D expenditure we used figures published by Chakma *et al* (2).

For R&D expenditure on specific poverty-related and neglected diseases, we used data provided by G-FINDER to us (personal communication by Dr Mary Moran, 24.02.2014). An aggregated version of the G-FINDER data set we used is available publicly at:
https://g-finder.policycures.org/gfinder_report/search.jsp (Last accessed: 15.03.2014)

G-FINDER publishes disease-specific expenditure data, but also data on R&D projects which are aimed at poverty-related and neglected diseases, but which cannot be linked exclusively to any one single disease, either because they aim at certain disease groups as a whole, or at non-disease-specific platform technologies (3). We excluded this data in our disease-specific analysis, making numbers smaller than they really are. However, we did include this non-disease-specific G-FINDER data in our summary analysis for the category "All poverty-related and neglected diseases (G-FINDER definition)".

We used G-FINDER figures for the financial years 2008-2012, notated in nominal 2007 US$.

Inflation adjustments

Values in US$ for years other than 2010 were adjusted to US$ 2010 price levels with the National Institutes of Health Biomedical Research and Development Price Index provided at:
http://officeofbudget.od.nih.gov/gbiPriceIndexes.html (Accessed 01.03.2014)

It should be noted that the use of the National Institutes of Health Biomedical Research and Development Price Index is controversial (4). In particular, we used it to adjust global R&D expenditure figures, even though it has been developed for the use with US R&D expenditure figures only. However, this approach still seems preferable to the alternative of using the implicit gross domestic product price index, or other economy-wide inflation adjustment measures (for a detailed discussion of these issues see (4) and the authors' reply contained herein).

G-FINDER only includes data on R&D investments specifically targeted at developing-country R&D needs. This implies that for certain diseases, only specific subtypes or strains, or specific target products (i.e. fixed-dose combinations and paediatric formulations in the case of HIV) are considered neglected, and are thus included in the G-FINDER data (3). It would therefore be methodologically consistent to compare G-FINDER R&D expenditure data with data on DALYs occurring in low and middle income countries (LMIC) only, in order to match disease definitions across the two categories most closely. However, 98.6% of the global disease burden caused by the 26 G-FINDER diseases included in our analysis occurs in LMICs. The use of DALY data from LMICs only would therefore not change the results of our analysis of the R&D Gap significantly. Therefore, for clarity, we have used global (HIC and LMIC) disease burden data.

The G-FINDER disease group “bacterial pneumonia & meningitis, caused by streptococcus pneumoniae or neisseria meningitidis” was considered equivalent to the GBD 2010 disease groups “pneumococcal meningitis”, “pneumococcal pneumonia” and “meningococcal infection”. The G-FINDER disease “rheumatic fever” was considered equivalent to “rheumatic heart disease” in GBD 2010.

Uncertainty intervals given for the Neglect Factor and the Dollar/DALY metric are based on the 95% uncertainty intervals for the DALY figures included in GBD 2010 only, and do not include the considerable but unquantified uncertainty in the R&D expenditure figures.

The following formula were used:

$$Neglect\ Factor = \frac{Disease\ burden\ in\ DALYs\ (\%\ of\ the\ total\ global\ disease\ burden)}{R\&D\ expenditure\ (\%\ of\ total\ global\ health\ related\ R\&D\ expenditure)}$$

$$Neglect\ Factor\ (lower\ bound\ estimate) =$$

$$= \frac{Disease\ burden\ in\ DALYs\ (\%\ of\ the\ total\ global\ disease\ burden, lower\ bound\ estimate)}{R\&D\ expenditure\ (\%\ of\ total\ global\ health\ related\ R\&D\ expenditure)}$$

$$Neglect\ Factor\ (upper\ bound\ estimate) =$$

$$= \frac{Disease\ burden\ in\ DALYs\ (\%\ of\ the\ total\ global\ disease\ burden, upper\ bound\ estimate)}{R\&D\ expenditure\ (\%\ of\ total\ global\ health\ related\ R\&D\ expenditure)}$$

References for supplementary file 1 – Methodological annexe:

1. Murray CJL, Vos T, Lozano R, Naghavi M, Flaxman AD, Michaud C, et al. Disability-adjusted life years (DALYs) for 291 diseases and injuries in 21 regions, 1990?2010: a systematic analysis for the Global Burden of Disease Study 2010. The Lancet. 2012;380(9859):2197-223.

2. Chakma J, Sun GH, Steinberg JD, Sammut SM, Jagsi R. Asia's ascent--global trends in biomedical R&D expenditures. The New England journal of medicine. 2014;370(1):3-6.

3. Moran M, Guzman J, Chapman N, Abela-Oversteegen L, Howard R, Farrell P, et al. Neglected Disease Research and Development: The Public Divide. G-FINDER Policy Cures, 2013.

4. Young AJ, Terry RF, Røttingen J-A, Viergever RF. Global Biomedical R&D Expenditures. New England Journal of Medicine. 2014;370(25):2451-2.
